# Bioprospecting and mechanistic insights of *Trichoderma* spp. for suppression of *Ganoderma*-induced basal stem rot in oil palm

**DOI:** 10.3389/fnut.2025.1582047

**Published:** 2025-07-10

**Authors:** M. Amrutha Lakshmi, M. Indraja, Udai B. Singh, A. R. N. S. Subanna, G. K Challa, Ritu Mawar, W. P. Dauda

**Affiliations:** ^1^ICAR-Indian Institute of Oil Palm Research, Pedavegi, India; ^2^ICAR-National Bureau of Agriculturally Important Microorganism, Mau, India; ^3^ICAR-Central Arid Zone Research Institute, Jodhpur, India; ^4^Crop Science Unit, Department of Agronomy, Federal University Gashua, Gashua, Nigeria

**Keywords:** *Ganoderma* spp., *Trichoderma afroharzianum*, antifungal mechanism, growth promotion, molecular phylogeny

## Abstract

**Purpose:**

Basal stem rot (BSR), with *Ganoderma* spp. as the principal causative agent, is an important oil palm disease, leading to significant stand loss and reduced yield potential. The use of antagonistic fungi, particularly *Trichoderma* spp., offers a sustainable approach to disease suppression through hyperparasitism, antibiosis, and rhizosphere competence. However, strain-dependent variability in antagonistic potential necessitates the selection of the most efficacious isolates for integrated BSR management. Here we show that *T. afroharzianum* exhibits superior antagonism against *Ganoderma* spp., in dual culture, inverted plate assay as well as cellfiltrate assays.

**Methods:**

From 50 Trichoderma isolates screened, 12 highly mycoparasitic strains (>80% *Ganoderma* suppression) were selected. To enhance applicability under field conditions, the selected strains were further evaluated against co-occurring soil-borne pathogens commonly associated with oil palm decline.

**Results:**

*T*. *afroharzianum* exhibited hydrolytic enzyme secretion (chitinase, cellulase, and pectinase), solubilized key macronutrients, and suppressed multiple soil-borne phytopathogens including *Rhizoctonia solani, R. bataticola, Fusarium solani, Lasiodeplodia theobromae Colletotrichum gleosporoides* and *Curvularia lunata*. A tailored *Trichoderma* consortium achieved 61.94% disease suppression, reduced foliar and bole severity by 48.59 and 20.22%, respectively, and increased plant height (47.59 ± 2.52 cm) and shoot fresh weight (15.83 ± 0.80 g).

**Implications/conclusion:**

These findings establish *T. afroharzianum* as a promising biocontrol agent for BSR suppression through multiple mechanisms, including competitive exclusion and pathogen inhibition. The results support its potential for field deployment as part of an integrated, climate-resilient disease management strategy in oil palm cultivation.

## 1 Introduction

Oil palm (*Elaeis guineensis* Jacq.), commonly known as the “Golden Palm,” is a global most important oil-bearing crop, producing 4–6 tonnes of edible oil and 0.5 tonnes of kernel oil per hectare per year, which is 5–10 times higher compared with other oil crops (1). Palm oil use is rising worldwide, due to its rich content of palm olein, renowned for having an appropriate proportion of saturated to unsaturated fatty acids, which can lower LDL cholesterol, as well as its excellent antioxidant properties. BSR caused by *Ganoderma* spp. is a global threat to oil palm plantations, causing up to 500 million USD in annual losses. The disease raises grave concerns about food security due to its significant impact on the global edible oil supply (2). This issue is particularly significant for India, one of the world’s largest oil-consuming and oil-importing countries, with limited domestic palm oil production, only 0.45 million hectares yielding 0.33 million tonnes.^1^ Palm oil cultivation has been largely confined to Andhra Pradesh, Telangana, and Kerala, which account for 98% of production.^2^ However, the spread of BSR threatens the objectives of the National Mission on Edible Oils – Oil Palm (NMEO-OP), which aims to expand cultivation across diverse agro-climatic zones, increase production, and achieve self-sufficiency.

The early foliar symptoms, such as petiole breaking and skirting, are often ambiguous and may be mistaken for vapor pressure deficit, leading to their frequent oversight or misinterpretation. The definitive observable symptom is the appearance of basidiocarps, which signals 80% of fungal invasion and marks the penultimate stage of death. The pathogen is unique in several characteristics such as dual feeding behavior, latent infection and prolonged gestation period (3, 4). Moreover, the persistent and recalcitrant soil-borne characteristics facilitate the production of an immense inoculum load including resistant forms such as mycelium, basidiospores, chlamydospores, and pseudo sclerotia, elevate it to the status of an exceptionally challenging pathogen to manage (5). The current sanitation and chemical management of BSR can only delay the progression of *Ganoderma* infection and prolongs the productive lifespan of infected palms. Since there are no seasonal breaks, the oil palm’s perennial and monoculture characteristics allow for the build-up of sizable inoculum and disease pressure reservoirs, which exacerbates the situation even more. This demands frequent and labour-intensive pesticide applications, which may prompt to the build-up of pesticidal resistance and toxic accumulation of residues, resulting in economic strain, health hazards and ecological damage. Moreover, they are not long lasting and inefficient in reducing inoculum load under field conditions (6, 7). Hence, curative strategies for managing this formidable pathogen remain largely impractical.

Under such circumstances, adoption of eco-friendly, and sustainable management measures, is the only viable option. As there is no resistant germplasm available for BSR, microbe-based biocontrol strategies evolved as a potential green tool for combating BSR in the era of sustainable agriculture. Studies have demonstrated remarkable results when microbial antagonists, including species of *Penicillium, Pseudomonas, Aspergillus, Bacillus, Streptomyces,* and *Trichoderma* were used individually or in consortia to manage BSR in plantations (5, 7–14). *Trichoderma* has emerged as a potent biocontrol agent against *Ganoderma*, primarily through mechanisms such as mycoparasitism, production of antifungal metabolites, and competitive exclusion, effectively limiting pathogen growth and spread. Its efficacy has been demonstrated in both field and green house environmental conditions (5, 7, 11, 12, 15–17).

**Figure 1:**
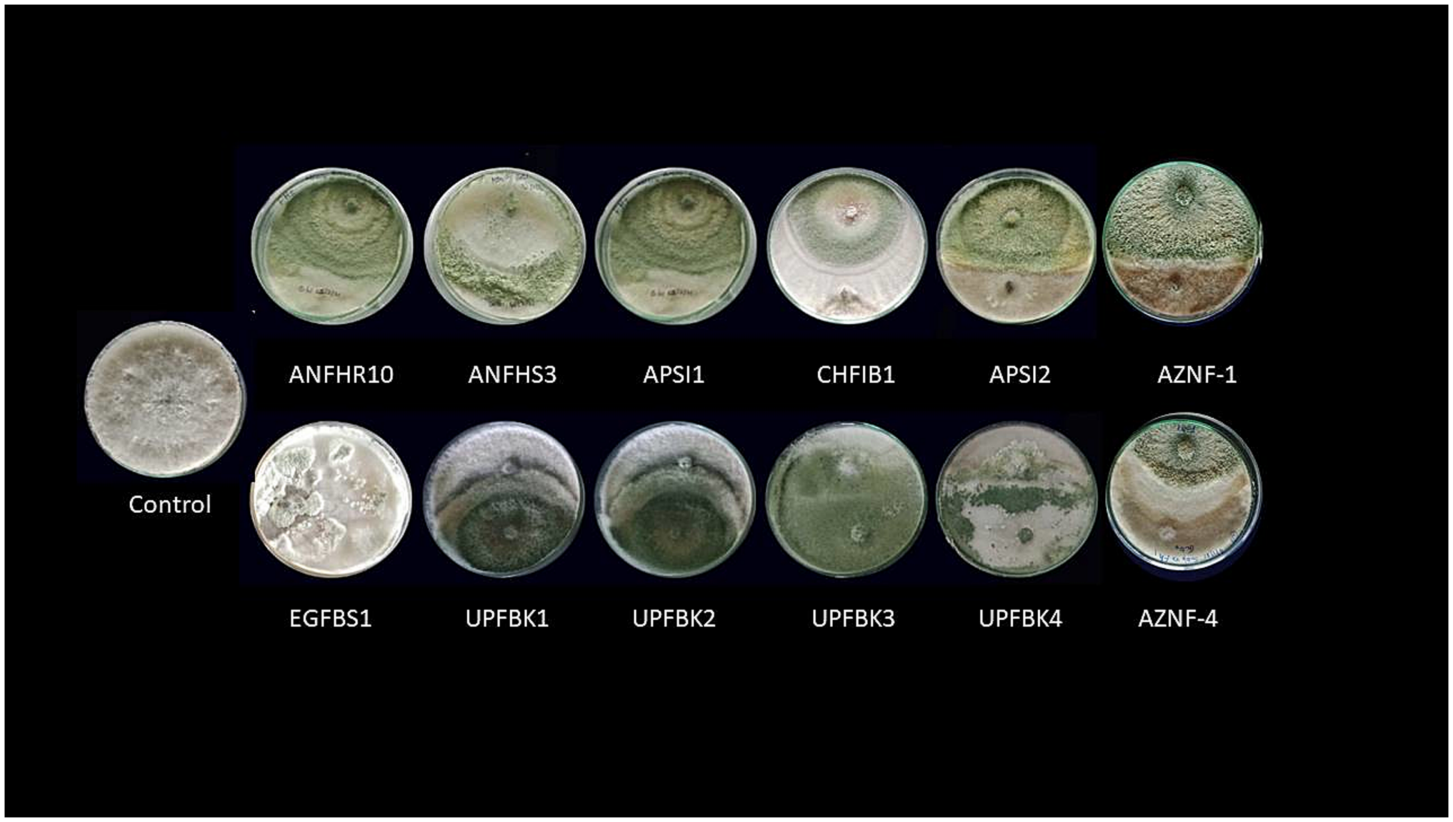
Antifungal efficacy of *Trichoderma* spp. against mycelial growth of *Ganoderma ellipsoideum* by dual plate technique.

The effectiveness of *Trichoderma* can vary significantly due to their unique feature of adapting to specific soil types, methods, and application timing, rhizospheric competency, abiotic and biotic factors. These variations pose a challenge to their biological stability, feasible delivery, and ease of commercialization for widespread field applications (18). Isolation of naturally occurring antagonistic *Trichoderma* strains from diverse and extreme niches is a promising strategy to obtain abiotic stress-tolerant antagonists, enabling the development of robust formulations tailored for application across varied climatic zones, ensuring consistent and effective BSR management (19). Hence, in the context of changing climate scenario, use of these diverse strains with compatible biocontrol activity can be successfully included in designing cocktails of bioagents instead of sole application of native strain to achieve consistent field performance in different soil environments and agricultural ecosystems (20). Despite the widespread use of *Trichoderma* as an agent of biological control, there remains a significant gap in the understanding its exploitation, characterization, and specific mode of action within the *Ganoderma*-oil palm system in India. Additionally, the opportunity to isolate stress-tolerant *Trichoderma* strains from India’s varied agroclimatic regions remains largely untapped. Selecting *Trichoderma* isolates from diverse agroclimatic regions allows for the identification of ecologically adapted strains with biocontrol efficacy, environmental resilience, and compatibility with variable soil–climate systems. This approach is particularly relevant for oil palm cultivation, which under the National Mission on Edible Oils–Oil Palm (NMEO-OP), is being expanded into non-traditional and climatically diverse regions across India. The deliberate inclusion of ecologically contrasting zones, ranging from arid zones (e.g., Rajasthan), humid tropical coasts (e.g., Kerala), to subtropical alluvial plains (e.g., Uttar Pradesh), was designed to capture the natural diversity of *Trichoderma* populations shaped by their native environments. Isolates from such varied contexts are more likely to exhibit stress tolerance, robust rhizosphere competence, and broad-spectrum antagonism traits critical for consistent performance in field conditions and for the development of climate-resilient biocontrol formulations. Given the foregoing, the current study aims to bio prospect *Trichoderma* spp. from diverse agroecological zones, characterize their biocontrol and growth promotion properties, and identify the most potent strains for managing BSR in oil palm contributing to sustainable disease management solutions (Figure 1).

## 2 Materials and methods

### 2.1 Test pathogen source and maintenance

The test pathogen used in this study was *Ganoderma ellipsoideum* (strain ITCC9346), obtained from the Indian Type Culture Collection (ITCC), Division of Plant Pathology, ICAR–Indian Agricultural Research Institute, New Delhi. Potato Dextrose Agar (PDA) was used as the growth medium. Sterile PDA plates and slants were prepared under aseptic conditions (Materials). The pathogen was sub-cultured on PDA and incubated at 28 ± 2°C. Mycelial growth was maintained on PDA slants and stored at 4°C for further experimental use (Methods).

### 2.2 Survey and isolation of *Trichoderma*

Soil, bark, root, and basidiocarp samples were collected from oil palm rhizospheres and decaying organic matter in five agroecological zones of India: Andhra Pradesh (East Coast Plains and Hills zone- humid to subhumid), Telangana (Southern Plateau and Hills- semi-arid to subhumid), Kerala (West Coast Plains and Ghats-Tropical), Andaman and Nicobar (Coastal Islands zone-Tropical) and Uttar Pradesh (Upper Gangetic Plains Region- subtropical). Samples were collected using clean polyethylene bags and transported to the laboratory. Trichoderma Specific Medium (TSM) was used for isolation, following the protocol of Papavizas and Lewis (21). All media, glassware, and tools were sterilized before use. Pure cultures were preserved in 20% glycerol at −20°C (Materials). A total of 150 samples were collected and stored at 4°C prior to processing. Soil samples were serially diluted (10^−3^ to 10^−6^) using sterile water and plated on TSM. Root and bark pieces were surface sterilized and directly plated onto TSM plates. Plates were incubated at 28 ± 2°C for 4 days. Colonies with typical *Trichoderma* morphology were purified using hyphal tip isolation. Purified cultures were stored in 20% glycerol for long-term preservation.

### 2.3 Preliminary screening of mycoparasitism by dual confrontation assay

Fifty new *Trichoderma* isolates, along with previously collected strains of *T. longibrachiatum* (NAIMCC-F-04134) and *T. harzianum* (NAIMCC-1723) from ICAR–Central Arid Zone Research Institute (CAZRI), Jodhpur, and *T. asperellum* and *T. harzianum* from ICAR–Indian Institute of Oil Palm Research (IIOPR), Pedavegi, were evaluated for *in vitro* mycoparasitism against *Ganoderma* using the dual-plate culture method (22, 23). Inoculum plugs of *Trichoderma* and *Ganoderma* were placed opposite each other, 1 cm away from the periphery of a 9 cm Petri dish, to allow clear observation of their antagonistic interaction. The reference strains are deposited at the National Agriculturally Important Microbial Culture Collection (NAIMCC), ICAR–National Bureau of Agriculturally Important Microorganisms (NBAIM), Mau, Uttar Pradesh, India. The plates were subsequently incubated at 28 ± 2°C until the mycelial growth fully covered the surface of the control plates. To verify the findings, the trial was conducted in three replications and repeatedly. The radial growth of *Ganoderma* (T) towards *Trichoderma* isolates was documented and the percentage inhibition of radial growth (PIRG) was determined viz.:


PIRG=[C−T/C]×100

Where the radial growth of *Ganoderma* mycelia in treatment and control plates were tagged as T and C, respectively. Based on results, the *Trichoderma* isolates were categorized into various groups. The top 12 potent isolates, demonstrating over 80% suppression of *Ganoderma,* were chosen for downstream investigation (Table 1).

**Table 1:** Potent *Trichoderma* isolates used in the study.

S. no.	Isolate designation	GPS location	Source	Place	Species identified/closest species match	Accession number*	Accession number^#^	Mean ± SD PIRG
1	AZNF1	26°14′33.8”N 73°00′23.4″E	Soil	Jodhpur, Rajasthan	*T. longibrachiatum*	NR120298.1	NAIMCC-F-04134	87.00 ± 0.0^g^
2	AZNF4	26°14′33.8”N 73°00′23.4″E	Soil	Jodhpur, Rajasthan	*T. harzianum*	NR134419	NAIMCC-1723	85.00 ± 0.0^i^
3	UPFBK1	25°53′50.9”N 83°29′19.6″E	Bark	Mau, Uttar Pradesh	*T. atroviridae*	OR244379	ITCC9393	97.67 ± 0.58^b^
4	UPFBK2	25°53′50.9”N 83°29′19.6″E	Bark	Mau, Uttar Pradesh	*T. asperellum*	OR244377	ITCC9355	99.77 ± 0.22^a^
5	UPFBK3	25°53′50.9”N 83°29′19.6″E	Bark	Mau, Uttar Pradesh	*T. afroharzianum*	OR244373	ITCC 9395	99.77 ± 0.40^a^
6	UPFBK4	25°53′50.9”N 83°29′19.6″E	Bark	Mau, Uttar Pradesh	*T. asperellum*	OR24478	ITCC9356	99.77 ± 0.23^a^
7	APSI1	16.8143° N 81.1254° E	Soil	Pedavegi, Andhra Pradesh	*T. asperellum*	–	–	89.00 ± 0.0^f^
8	APSI2	16.8143° N 81.1254° E	Soil	Pedavegi, Andhra Pradesh	*T. harzianum*	–	–	86.00 ± 0.87^h^
9	ANFHR10	11.613764 92.716515	Roots	Port Blair, Andaman and Nicobar	*T. virens*	OR244374	ITCC9357	96.00 ± 0^c^
10	ANFHS3	11.613764 92.716515	Soil	Port Blair, Andaman & Nicobar	*T. longibrachiatum*	OR244375	–	94.18 ± 0.41^d^
11	CHFIB1	17.0688° N 80.9838° E	Bracket	Chintalapudi, Andhra Pradesh	*T. virens*	OR24476	ITCC9358	90.83 ± 0.50^e^
12	EGFBS1	17.0005° N 81.8040° E	Bracket	Rajahmundry, Andhra Pradesh	*T. cremeum*	OR244380	ITCC 9394	96.50 ± 0.05^c^
	CD (0.05)							0.645
	CV (%)							0.41

PIRG, Percentage Inhibition of Radial Growth; SD, Standard Deviation; In a column, means followed by a common letter(s) are not significantly different by LSD (*p* = 0.05): Accession Number*: Sourced from NCBI GenBank Accession Number^#^: Indian Type Culture Collection Centre, New Delhi and National Agriculturally Important Microbial Culture Collection, Uttar Pradesh, India.

### 2.4 Evaluation of antibiosis activity of *Trichoderma* isolates

#### 2.4.1 Inverted plate assay for determining volatile antibiosis

**Figure 2:**
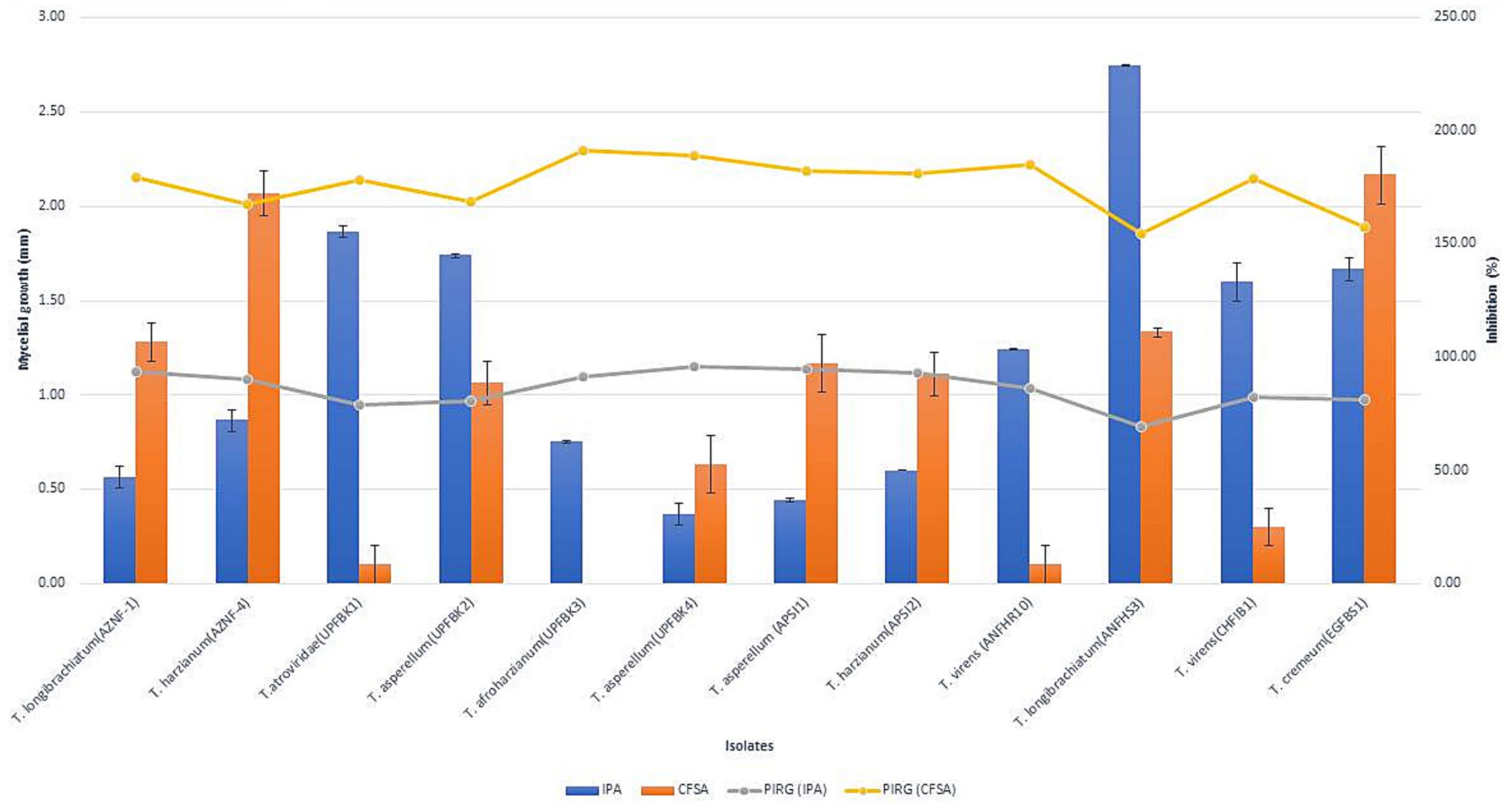
Inhibition of *Ganoderma ellipsoideum* growth by antifungal volatiles and non-volatiles from *Trichoderma* species. The graph shows the effect of *Trichoderma*-derived volatiles (bars) and non-volatiles (line) on *Ganoderma ellipsoideum* growth inhibition. Data represent percentage growth reduction, with higher concentrations or exposure times leading to greater inhibition. Statistical significance is denoted by asterisks (*p < 0.05, **p < 0.01).

A fifteen milliliter amount of 2.4% (w/v) PDA was added to 90 mm Petri dishes for an inverted plate assay (IPA). A mycelial disc with 5 mm in diameter was moved from the *Trichoderma* culture’s periphery to the middle of medium. Additionally, a mycelial disc was inverted over the *Trichoderma* culture after being moved from the *Ganoderma* culture’s periphery to a fresh Petri dish. For 7 days, the assembled plates were kept at 25°C in the dark after being sealed using parafilm and then wrapped with three layers of plastic wrap to ensure minimal gas exchange and containment of volatile compounds. For the control, only *Ganoderma* was inoculated in the lower Petri dish, while the negative control consisted of uninoculated medium (24). The volatile-based inhibition of *Ganoderma* by various *Trichoderma* isolates was evaluated based on PIRG, as described earlier (Figure 2).

#### 2.4.2 Cell filtrate assay for evaluating soluble non-volatile antibiosis

The cell filtrate assay was employed to assess the impact of non-volatiles on the growth and dry weight of test pathogen, using both solid and liquid poisoned food assay (CFSA and CFLA). One mL of each *Trichoderma* conidial suspension was mixed with 100 mL of Potato Dextrose Broth (PDB) to make the culture filtrate. The resultant mixture was then incubated for 7 days in an orbital shaker at 28°C and 100 rpm. Following a 15-min centrifugation at 10,000 rpm at 4°C, the culture was filtered through syringe filters with 0.22 μm size to remove the cell-free supernatant. A 5 mm disc of *G. ellipsoideum* was used to inoculate the plates for the agar experiment, which involved adding five milliliters of culture filtrate to molten PDA. After all of the mycelia had covered the control plate (which did not include culture filtrate), the pathogen’s radial mycelial growth was measured. The PIRG was determined as explained previously. For the broth assay, 10 mL of cell filtrate was added to 50 mL of PDB, followed by inoculation with a 5 mm *Ganoderma* mycelial disc. The co-incubated cultures were maintained in an orbital shaker at 100 rpm and 28°C for 7 days. The *Ganoderma* mycelial mat was separated using pre-weighed Whatman No. 1 filter paper and subsequently dried at 50°C for 4 h in a hot air oven. The reduction in dry weight was calculated relative to the *Ganoderma*-inoculated control (without *Trichoderma* culture filtrate). The negative control consisted of uninoculated broth without *Trichoderma*. All treatments were performed in triplicate.

### 2.5 Morphological characterization

The morphological and cultural features of *Trichoderma* isolates were examined on PDA medium using the protocol outlined by Samuels et al. (25). Microscopic observations were conducted based on the monograph by Rifai (26). A 5 mm mycelial disc of each *Trichoderma* isolate (cultured for 7 days) was inoculated on the periphery of the Petri dishes and incubated at 28 ± 2°C for 1 week. The radius of the colony was determined at 24, 48 and 72 h. The growth rates were calculated by averaging the measurements from three independent trials, each conducted in triplicates. The colony characteristics, including shape, texture, growth patterns, colony margins and presence of concentric rings, were recorded. Pigmentation on both colony surface and reverse side of the plate was also noted. The microscopic observations were made following the guidelines in Rifai (26), focusing on the arrangement and Conidiophores branching pattern, conidia size and form, and the nature of the spores were observed using Leica DM 5000 B microscope (Leica MikrosystemsVertrich GmbH, Germany).

### 2.6 *Trichoderma* spp. molecular identification and bioinformatics analyses

Monosporic cultures of *Trichoderma* strains were molecularly identified. Agar plugs (10 mm) from 7-day-old cultures were inoculated to PDB and an actively growing mycelial mat (150 mg) was harvested on the 10^th^ day and blot-dried on sterile filter paper. DNA extraction from *Trichoderma* mycelia was performed using HiPurA® Fungal DNA Purification Kit (HiMedia, India). NanoDrop™ Spectrophotometer ND-1000 (Eppendorf, Germany) and agarose gel electrophoresis (0.8%) were used to probe the purity status and the concentration of the isolated DNA. Pure DNA was then amplified via PCR amplification using Internal Transcribed Spacer primers: ITS1 (5′ -TCC GTA GGT GAA CCT GCG G- 3′) and ITS4 (5′ -TCC TCC GCT TAT TGA TAT GC-3′) and procedures developed by White et al. (27). The PCR composition was standardized as follows: 1.5 μL of 10x PCR buffer (HiMedia, India), 0.15 μL of 10 mM dNTP (HiMedia, India), 1.5 μL (10 pg./μL) each of both forward reverse primers and 0.15 μL of 5 U Taq polymerase (HiMedia, India) and made to a reaction volume of 15 μL by adding 10.275 μL of nuclease free Milli-Q water. PCR conditions for primers were standardized as 94°C – 3 min, 94°C –30 s, 51°C –30s, 72°C –1.0 min; finally 72°C–10 min. To the amplified product, 2.0 μL Ethidium Bromide dye (Thermo Fisher, USA) was added, and PCR products (5 μL/sample) were electrophoresed in a 1.2 per cent agarose gel for 45 min. In a gel documentation system (IGENE LABSERVE, India), the gel was analyzed under ultraviolet light after being stained with ethidium bromide. The amplicons were sent for Sanger sequencing. Sequences with the accession number shown in Table 1 were added to GenBank. To find related sequences, the consensus sequence was BLAST-searched against the NCBI database where the top 10 sequences were chosen based on the maximum identity scores for downstream analyses. The Clustal-W algorithm was employed to run the Multiple DNA sequence alignments. Evolutionary analyses were conducted using the concatenated 5′- ITS region (575 bp), with reference sequences corresponding to the most closely related taxa downloaded from GenBank. The Maximum Composite Likelihood method was used to measure the evolutionary distances, expressed as number of base substitutions per site implemented in the PhyML program, with the tree topology further evaluated by bootstrap resampling of 1,000 iterations (28). The neighbor–joining approach was used to estimate the evolutionary history (29), and a bootstrap study with 1,000 repetitions was conducted to evaluate the tree topology’s robustness (30). MEGA X was used for all evolutionary analyses (31) (Table 2).

**Table 2:** Antibiosis efficacy of *Trichoderma* spp. against *Ganoderma ellipsoideum*.

Treatment	Isolates	Per cent inhibition in inverted plate assay	Per cent inhibition in cell filtrate solid assay	Per cent inhibition in cell filtrate liquid assay
1	*T. longibrachiatum* AZNF-1	93.70 (9.68) ± 0.64^b^	85.74 (9.26) ± 1.08^ef^	60.74 (7.79) ± 1.28^efg^
2	*T. harzianum* AZNF-4	90.37 (9.51) ± 0.64^d^	77.04 (8.78) ± 1.28^g^	57.04 (7.55) ± 2.57^g^
3	*T. atroviridae* UPFBK1	79.26 (8.90) ± 0.32^h^	98.89 (9.94) ± 1.11^ab^	72.59 (8.52) ± 1.28^bc^
4	*T. asperellum* UPFBK2	80.67 (8.98) ± 0.11^g^	88.15 (9.39) ± 1.28^d^	68.15 (8.26) ± 1.28^cd^
5	*T. afroharzianum* UPFBK3	91.63 (9.57) ± 0.06^c^	100.00 (10.00) ± 0.00^a^	82.22 (9.07) ± 3.85^a^
6	*T. asperellum* UPFBK4	95.93 (9.79) ± 0.64^a^	92.96 (9.64) ± 1.70^c^	65.93 (8.12) ± 1.28^de^
7	*T. asperellum* APSI1	95.07 (9.75) ± 0.13^a^	87.04 (9.33) ± 1.70^def^	65.19 (8.07) ± 1.28^def^
8	*T. harzianum* APSI2	93.33 (9.66) ± 0.00^b^	87.67 (9.36) ± 1.28^de^	67.41 (8.21) ± 1.28^cd^
9	*T. virens* ANFHR10	86.19 (9.28) ± 0.06^e^	98.89 (9.94) ± 1.11^ab^	68.89 (8.30) ± 0.00^cd^
10	*T. cremeum* EGFBS1	69.48 (8.34) ± 0.06^i^	85.19 (9.23) ± 0.26^f^	64.44 (8.03) ± 2.22^def^
11	*T. virens* CHFIB1	82.22 (9.07) ± 1.11^f^	96.67 (9.83) ± 1.11^b^	78.52 (8.86) ± 3.39^ab^
12	*T. longibrachiatum* ANFHS3	81.48 (9.03) ± 0.64^f^	75.93 (8.71) ± 1.70^g^	60.00 (7.73) ± 10.18^fg^
CD (0.05)		0.04	0.11	0.37
CV		0.28	0.71	2.67

Figures in the parentheses were square root transformed; in a column, means followed by a common letter(s) are not significantly different by LSD (*P* = 0.05).

### 2.7 Evaluation of broad-spectrum efficiency

Dual confrontation assays of 12 *Trichoderma* isolates were performed against oil palm-associated pathogens at nursery and field levels. The targeted pathogens included *Curvularia lunata* (leafspot), *Collectotrichum gleosporoides* (anthracnose), *Lasiodeplodia theobromae* (leaf blight), *Rhizoctonia bataticola, Rhizoctonia solani* causing (leaf rot), and *Fusarium solani* (bunch rot) at nursery and field level, respectively. The mycoparasitism was assessed based on PIRG (%), as previously described.

### 2.8 Qualitative assessment of detoxifying and lytic enzyme synthesis

The qualitative evaluation of detoxifying and lytic enzyme production was conducted using 7-day-old cultures of *Trichoderma*, with each assay performed in triplicate following the procedure of Cappuccino and Sherman (32). For every biochemical test, a non-inoculated plate served as the negative control. The observation of a distinct halo surrounding the colony after 7 days of incubation indicated positive lytic activity, except for catalase activity, which was confirmed by the immediate formation of bubbles, corresponding to oxidative stress tolerance. As explained by Saadaoui et al. (33), catalase activity was assessed by inserting (5 mm diameter plug) an actively developing *Trichoderma* cultures on a glass slide in a Petri dish plate, and then applying 3% hydrogen peroxide. The production of protease enzyme was detected by growing *Trichoderma* strains on Glucose–Yeast–Peptone medium enriched with 1% skim milk (pH 6.5), as outlined by Mahfooz et al. (34). The strains of *Trichoderma* were inoculated on GYP medium which was supplemented with 1% soluble starch in order to assess *α*-amylase activity (35). Plates were then incubated for 7 days before being flooded for 10 min with a 1% iodine solution in potassium iodide (2%), followed by the removal of the solution. Chitinase production was evaluated as per López et al. (36) by culturing *Trichoderma* strains on a basal media containing colloidal chitin, prepared using the method of Roberts and Selitrennikoff (37). Cellulase production was estimated by inoculating a 5 mm *Trichoderma* disc onto a basal medium with 0.1% carboxymethylcellulose (CMC) and incubating for 5 days at 28 ± 2°C (38). A 0.01% concentration of Congo red solution was then added for 15 min, and rinsed with 1% NaCl for 5 min. Finally, pectinase production was evaluated by placing a 5 mm *Trichoderma* disc on a medium containing 1% pectin and then incubated for 5 days at 28 ± 2°C, followed by the application of Gram’s iodine solution to the pectin agar (39).

### 2.9 Bioassays for plant growth promoting (PGP) qualities

The synthesis of various PGP characteristics, including IAA, GA, NH_3_, HCN, and siderophore, was assessed in the 12 *Trichoderma* isolates that made the short list. The experiments were conducted twice, and each assay was run with three replications.

#### 2.9.1 Colorimetric detection of indole-related compounds

Using the Salkowski test, the *in vitro* synthesis of IAA for each *Trichoderma* strain that promotes plant growth was identified (40). Each culture was initiated by inoculating 10 mL of PDB supplemented with 0.1% L-tryptophan with two 5 mm plugs of a 7-day-old *Trichoderma* strain. They were then shaken for 72 h at 100 rpm at 28.0 ± 2.0°C. After centrifuging 1.5 mL of culture for 10 min at 10,000 rpm, the supension was extracted, and 1 mL of it was then added in duplicate to 2.0 mL of Salkowski reagent. The optical density was taken at 530 nm following a 30 min dark incubation period at room temperature (41). A red appearance suggested that the fungal strain was producing IAA. A standard graph made using known amounts of pure IAA was used to compute the quantity of IAA emitted. A UV–vis spectrophotometer (Simadzu, Japan) was used to detect the color intensity at 530 nm in order to determine IAA. The number of indole-related substances was ascertained using a standard curve that was made by suspending IAA in ethanol (100%) at a concentration of 1 mg/mL and then diluting it in PDB medium to a concentration of 1–10 μg/mL. PDB and L-tryptophan served as controls. Three biological replicates were used in the experiment. Linear Regression (R2 = 0.998, *p* < 0.0001) was used to infer the measured values for each strain.

#### 2.9.2 Colorimetric detection of gibberellic acid (GA)

Each 5-day-old *Trichoderma* spp. disc, measuring 5 mm, was infected separately in 100 milliliters of PD broth and cultured for a week at 28 ± 2°C. Following incubation, the amount of GA in the Trichoderma culture filtrate was measured using a colorimetric assay with 0.5 M potassium ferricyanide and 0.5 M zinc acetate reagents, as previously mentioned. The absorbance of the resultant solution was determined using the Mahadevan and Sridhar (42) method at 254 nm in a UV–Vis spectrophotometer (Simadzu, Japan). Based on the standard graph created from known gibberellin concentrations, the gibberellin concentration was computed in μg/mL.

#### 2.9.3 Qualitative detection of ammonia

Cappuccino and Sherman's (32) colorimetric approach was used to measure ammonia production. In short, 10 mL of peptone water was mixed with two plugs of 5 mm week old actively growing *Trichoderma* and shaken for 7 days at 25/28°C. One milliliter of each culture supernatant was mixed with 1.0 mL of Nessler’s reagent (Himedia), which contains 7% KI, 10% HgCI_2_, and a 50% aqueous solution of NaOH (32%). Ammonia is released when a yellow to brown precipitate forms.

#### 2.9.4 Qualitative hydrogen cyanide (HCN) production

The method used to estimate HCN production was adapted from Kloepper et al. (43). A solid PDA mixed with 4.4 g/L of succinate or glycine was used to cultivate *Trichoderma* spp. White filter paper discs that were cut to the same size as the Petri dish’s top lid were carefully placed on the lid of each plate after being submerged in an alkaline solution of picric acid (0.5% v/v and 1.25% w/v sodium carbonate) in water. After sealing the plates with Parafilm, they were incubated at 28 ± 2°C for 7 days. Following incubation, the filter paper’s color changed from yellow to light brown or reddish brown, indicating the synthesis of HCN (44).

#### 2.9.5 Mineral solubilization assay

For each solubilization test, the respective medium was inoculated with a 5 mm and week old *Trichoderma* culture, with three repetitions per strain on each plate, followed by incubation for a week at 28 ± 2°C. Insoluble tricalcium phosphate medium from the National Botanical Research Institute was used to examine the phosphate solubilization ability; activity was demonstrated by the formation of a distinct halo surrounding the colony during incubation (45). Potassium solubilization was assessed using Aleksandrov medium containing potassium aluminium silicate, with a clear zone around the colony signifying solubilization (46). A modified Pikovskaya medium enriched with zinc oxide was used to examine the potential for zinc solubilization; the creation of a clear zone suggested zinc solubilization (47). Spot cultures on Chromeazurol S (CAS) media were used to measure siderophore production; colonies with yellow or orange halos surrounding them were considered to be producing siderophores (48). These observations determined the solubilization or production capabilities of the *Trichoderma* strains.

### 2.10 Assessment of prospective *Trichoderma* strains in a greenhouse for the suppression of BSR disease in oil palm seedlings

#### 2.10.1 Planting material, inoculum preparation, and inoculation

Oil palm sprouts obtained from the Seed Processing Unit (ICAR–IIOPR, Pedavegi) were surface sterilised. While planting, sprouts of oil palm (IOPPVDD000001) with differentiated plumule and radicle were placed in a shallow pits 2–3 cm depth and covered with soil to 10 mm depth. Polyethylene bags (23×15 cm of 2 kg capacity with 250 gauge) were filled with sieved soil (<2 mm). After sterilizing the soil with a liquid cycle in an autoclave (121°C, 20 min, 100 kPa), it was left to cool for 2 days at room temperature. It was sterilized a second time under same conditions before potting. Further, plants were kept in a net house at 26 ± 3°C (light/dark), with a photoperiod of 16 h and >70% relative humidity. Plants were irrigated with tap water daily, fertilized once a week and green house sanitation maintained.

A total of five *Trichoderma* isolates (*T. longibrachiatum* AZNF1, *T. atroviride* UPFBK1, *T. asperellum* UPFBK4, *T. afroharzianum* UPFBK3, and *T. virens* ANFHR10) were evaluated for their bioinoculant potential, both individually and in consortium, against *Ganoderma ellipsoideum*. For the *in planta* study, strains were carefully selected based on their strong antagonistic activity, plant growth-promoting traits, and thermotolerance to ensure compatibility and effectiveness under variable conditions. To 100 mL of PDB, one mycelial disc (5 mm) was inoculated and incubated for 7 days at 28°C in an orbital shaker at 100 rpm. Mycelial mats were collected aseptically and blended with a sterilized hand-held blender (Philips®HL1655/00) for 3 min. The conidial strength was calculated using a haemocytometer by serial dilution method. A total 100 mL of each spore suspension (6–7×10^6^ spores/mL) inoculum were drenched at the base of four-month-old oil palm seedling. Booster doses were provided 15 and 30 days after planting (DAP) by drenching 100 mL conidial suspension. Inoculum of *Ganoderma* was prepared by inoculating mycelial disc (10 mm) in 100 mL PDB and incubated for 4 days at 28°C in orbital shaker at 100 rpm. Mycelial mats were then aseptically harvested and blended for 3 min with 0.002% Tween® 20 in a sterilized hand-held blender (Philips®HL1655/00). The final mycelial concentration was adjusted to 10^5^ fragments/mL using a haemocytometer. Seven days post *Trichoderma* treatment, *Ganoderma* inoculation was done by mycelial root immersion method following Purnamasari et al. (49) with slight modifications. Seedlings were uprooted carefully, immersed mycelium suspension for 30 min and replanted back in *Trichoderma* treated soil.

#### 2.10.2 Assessment of BSR disease severity

Disease was assessed and scored following a two-week incubation period. A scale of 0–4 was used to score the disease based on the symptoms that were seen; 0 represented healthy plants, 1 represented mildly chlorotic patches on the leaves, 2 represented yellowing in 1–2 leaves, 3 represented yellowing on numerous leaves (> 2 leaves), and 4 represented dead plants (15). The following formula was used to measure the severity of bole and root symptoms (%) and foliar symptoms (%) (17):


$$\frac{\mathrm{FS}\;(\%)=(a\times 1)\pm (b\times 0.5)\times 100}{c}$$

a = number of browned/wilted leaves; b = number of yellowed leaves, c = total number of leaves.


$$\mathrm{BRS}(\%)=\sum \frac{\text{Number of seedling in the rating}\times \text{rating number}\times 100}{\text{Total number of seedlings evaluated}\;x\;\text{high rating value}}$$

Where 0 = healthy; absence of internal rot, 1 = 20% plant tissue rot, 2 = 20–50% plant tissue rot, 3 = > 50% plant tissue rot.

The dry weights of the roots, shoots, and height were measured after 8 weeks. Four seedlings were selected randomly from each treatment throughout the trial. The height of the shoots was documented from the base to the top of the stem. The roots and shoots were oven-dried at 70°C until their weight remained constant. After then, each of them was weighed independently, and the results were noted.

### 2.11 Scanning electron microscopy (SEM)

SEM analysis was conducted following the method of Sreenayana et al. (50) using an FEI Quanta 250 (Czech Republic) to examine the morphological features of *Ganoderma ellipsoideum*, *Trichoderma afroharzianum* UPFBK3, their interactions, and colonization on oil palm seedling roots. Three types of samples were prepared: (i) pure cultures of *G. ellipsoideum* and the most potent *T. afroharzianum* UPFBK3 isolate, (ii) dual-culture interaction zones involving selected *T. afroharzianum* isolates based on *in vitro* antagonism assays, and (iii) root tissues from oil palm seedlings inoculated with *T. afroharzianum*. Samples were fixed in 2.5% glutaraldehyde and incubated it at 4°C for 24 h, dehydrated through a graded ethanol series (30, 50, 70, 90, and 100%), followed by critical point drying. The dried samples were mounted on aluminum stubs, sputter-coated with gold, and examined under SEM to assess fungal morphology, interaction zones, and colonization patterns on root surfaces.

### 2.12 Statistical analysis

Analysis of variance (ANOVA) was employed to analyze the data collected. Data in percentages were square root transformed, and mean values were compared using the least significant difference test. All analyses were performed using the Web Agri Stat Package 2.0, a web-based statistical package.

## 3 Results

### 3.1 Evaluation of the antagonistic feature of *Trichoderma* isolates

Fifty native *Trichoderma* strains, each exhibiting varying levels of antifungal activity against *Ganoderma* spp. were successfully isolated from diverse samples collected across multiple geographical locations of India, including 11 from Andaman and Nicobar, 5 from Telangana, 6 from Kerala, 12 from Uttar Pradesh, and 16 from Andhra Pradesh (Supplementary Table 1). Based on the inhibition (%) on radial growth, the *Trichoderma* strains were classified into the following categories: very strong (>90%) with 8 isolates, strong (80–90%) with 4 isolates, moderate (60–80%) with 21 isolates, weak (50–60%) with 9 isolates, and very weak (<50%) with 7 isolates. Overall, the antagonistic efficacy of the isolates against *G. ellipsoideum* varied significantly across dual culture, IPA, CFSA, and CFLA assays. Notably, UPFBK3 (97.77, 94.81, 100.00, 82.60%), UPFBK1 (97.77, 91.11, 98.44, 72.59%), and UPFBK4 (97.77, 95.93, 95.74, 71.11%) exhibited consistently high performance across all methods. In contrast, the least effective isolates were EGFBS1 (69.48, 85.19, 64.44%) followed by AZNF4 (85.00, 88.89, 75.93, 57.04%), which demonstrated significantly lower antifungal efficacy in all assays.

### 3.2 Morphological characterization of *Trichoderma* isolates

**Figure 3:**
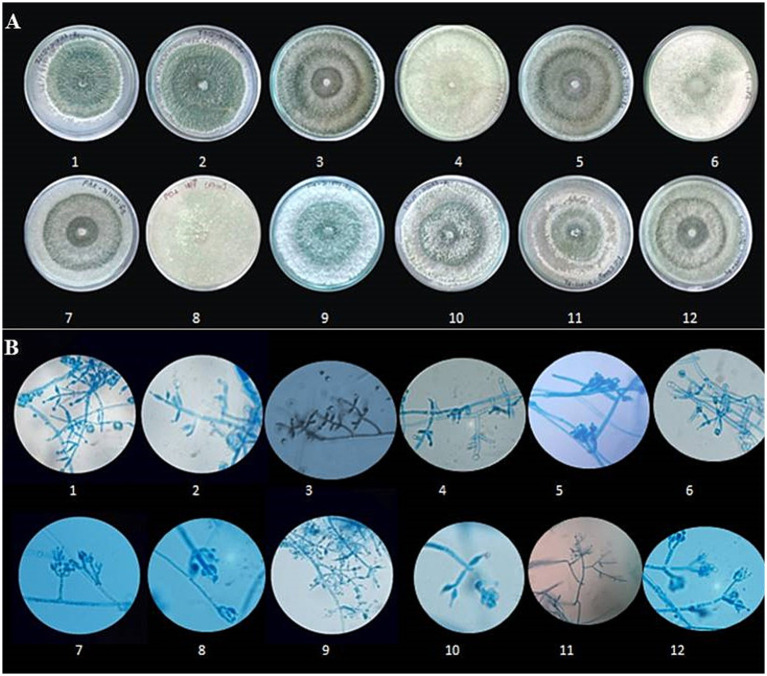
Morphological characterization of *Trichoderma* species. **(A)** Colony morphology of different *Trichoderma* isolates on culture media. Rows 1–12 show variations in colony texture, colour, and growth patterns among isolates. **(B)** Microscopic images of *Trichoderma* hyphae and conidia under a light microscope at 400 × magnification (Scale-100 μm). Rows 1–12 depict structural differences, including hyphal branching, spore formation, and conidial arrangement, highlighting the diversity among the isolates.

The morphological characterization of *Trichoderma* isolates revealed that the majority of colonies were circular with smooth margins. The texture of the colonies varied from smooth to fluffy to powdery with or without concentric rings. Initially, the colonies were white, transitioning to light green and eventually to dark green pigmentation due to production of conidia. Some colonies exhibited reverse pigmentation that ranged from yellow to brownish (Figure 3A). An earthy or musty odour was noted for most of the isolates. Microscopic examination showed that the conidiophores were short or elongated upright and smooth-walled, with branching patterns ranging from regular to irregular. Phialides were short or elongated flask-shaped and typically arranged in single, whorls or clusters. Conidia were observed to be ellipsoidal, subglobose, or ovoid. The hyphae were septate, branched, and ranged in colour from white to pale greenish (Figure 3B). All the 12 isolates belonging to seven species were differentiated detailed in Supplementary Table 2. The results of the study on radial mycelial growth at 48 h reveal statistically significant variations (CD = 0.39) (Table 3). Most strains achieved full growth (9 cm diameter) within 3 days, except for EGFBS1 (3.69 ± 0.01 cm) and AZNF4 (3.76 ± 0.01 cm), which exhibited significantly slower growth. Among the strains, UPFBK3 showed the highest growth at 48 h (3.58 ± 0.25 cm), followed closely by ANFHS3 (3.49 ± 0.44 cm). In contrast, AZNF4 demonstrated the slowest growth at 48 h, reaching only 2.46 ± 0.11 cm.

**Table 3:** Growth rate assay of *Trichoderma* isolates.

Treatment	Isolates	Radial mycelial growth in cm		
		24 h	48 h	72 h
1	*T. asperellum* APSI1	0.63 ± 0.14	2.74 ± 0.16^de^	4.50 ± 0.00^a^
2	*T. harzianum* APSI2	0.70 ± 0.04	3.04 ± 0.20^bcd^	4.50 ± 0.00^a^
3	*T. longibrachiatum* AZNF1	0.77 ± 0.21	3.47 ± 0.24^a^	4.50 ± 0.00^a^
4	*T. harzianum* AZNF2	0.58 ± 0.22	2.46 ± 0.11^c^	3.76 ± 0.01^b^
5	*T. atroviridae* UPFBK1	0.58 ± 0.22	3.38 ± 0.02 ^ab^	4.50 ± 0.00^a^
6	*T. asperellum* UPFBK2	0.70 ± 0.07	2.73 ± 0.07^de^	4.50 ± 0.00^a^
7	*T. afroharzianum* UPFBK3	0.65 ± 0.05	3.58 ± 0.25^a^	4.50 ± 0.00^a^
8	*T. asperellum* UPFBK4	0.63 ± 0.08	2.87 ± 0.04^cd^	4.50 ± 0.00^a^
9	*T. virens* ANFHR10	0.71 ± 0.28	3.24 ± 0.48^abc^	4.50 ± 0.00^a^
10	*T. longibrachiatum* ANFHS3	0.72 ± 0.14	3.49 ± 0.44^a^	4.50 ± 0.00^a^
11	*T.cremeum* EGFBS1	0.50 ± 0.05	2.67 ± 0.08^de^	3.69 ± 0.01^c^
12	*T. virens* CHFIB1	0.71 ± 0.08	3.53 ± 0.08^a^	4.50 ± 0.00^a^
CD (0.05)		NS	0.39	0.004
CV		23.53	7.39	0.54

In a column, means followed by a common letter(s) are not significantly different by LSD (*P* = 0.05).

### 3.3 Molecular identification of *Trichoderma* isolates

**Figure 4:**
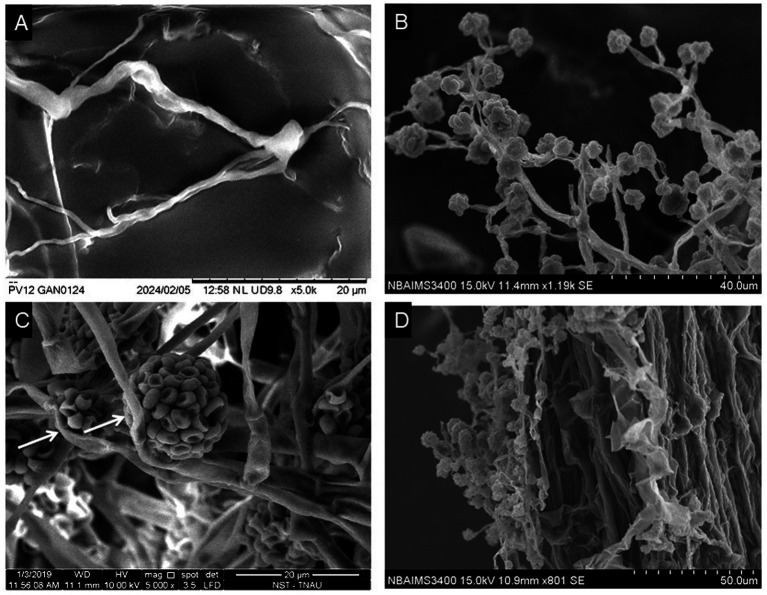
Scanning electron microscope (SEM) analysis of *Ganoderma ellipsoideum* and *Trichoderma afroharzianum* interactions. **(A)** Healthy tubular hyphae of *G. ellipsoideum* (magnification: 5,000×, scale bar: 20 μm). **(B)** Morphology of *T. afroharzianum*, showing branched hyphae, flask-shaped phialides, and smooth-walled ellipsoidal to subglobose conidia (magnification: 5,000×, scale bar: 20 μm). **(C)** Hyperparasitism of *T. afroharzianum* UPFBK3, with arrows indicating damage on *G. ellipsoideum* hyphae (magnification: 8,000×, scale bar: 10 μm). **(D)** Root colonization by *T. afroharzianum*, demonstrating its interaction with the plant root surface (magnification: 5,000×, scale bar: 20 μm).

Using ITS1 and ITS4 primers, a PCR amplification of about 575 bp of the ITS region for eight native isolates was obtained, and forward and reverse sequencing were then performed to identify the putative strains (Supplementary Table 3). We were able to obtain sequences that have between 98 and 100 percent similarity to *Trichoderma* strains thanks to the NCBI database’s BLASTn alignment. The phylogenetic tree analysis results, as presented in Figure 4, reveal that the studied isolates were categorized into six distinct clades, each representing a specific species within the *Trichoderma* genus. Clade 1 contains two isolates, identified as CHFIB1 (OR244374) and ANFHR10 (OR244376), which were determined to be *T. virens*. The Clade 2 contains a single isolate, UPFBK3 (OR244373), which was identified as *T. afroharzianum*. Clade 3: Consists of the isolate EGFBS (OR244380), identified as *T. cremeum*. Clade 4 harbors the isolate ANFHS3 (OR244375), which was identified as *T. longibrachiatum*. Clade 5 includes two isolates, UPFBK2 (OR244377) and UPFBK4 (OR244378), both identified as *T. asperellum*. Clade 6 comprises the isolate UPFBK1 (OR244379), which was identified as *T. atroviride*. The ITS sequence and GenBank accession number are included in the summary of the sequence results that are shown in Supplementary Table 1.

### 3.4 Determination of hydrolytic and growth activities of *Trichoderma* isolates

**Figure 5:**
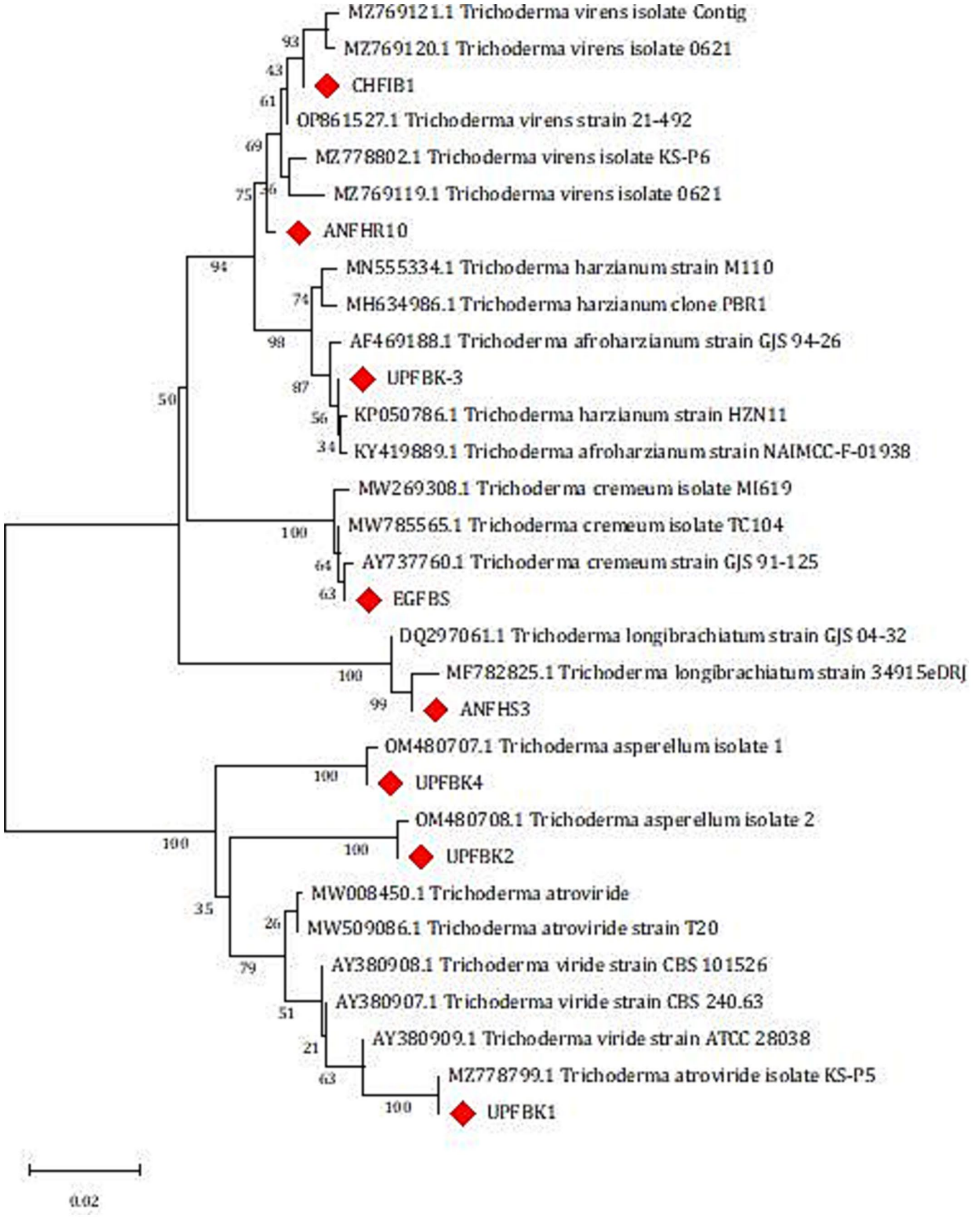
Phylogenetic analysis of *Trichoderma* isolates based on Internal Transcribed Spacer (ITS1 and ITS4) sequences using maximum likelihood analysis. Isolates in this study are highlighted with red diamonds. The scale bar represents genetic distance.

The hydrolytic and growth promotion enzyme activity results demonstrated significant variation among the tested *Trichoderma* isolates (Figure 5 and Tables 4, 5). *T. asperellum* APSI1 and *T. virens* CHFB1 emerged as the strongest enzyme producers, with ++++ activity in chitinase, cellulase, and pectinase, amylase activity and moderate catalase activity (+++). *T. asperellum* UPFBK4 and *T. afroharzianum* UPFBK3 followed closely, with ++++ activity in cellulase and pectinase, alongside strong chitinase production (+++). The weakest performers were *T. cremeum*EGFBS1, which exhibited low activity (+) in cellulase and pectinase, and only moderate catalase (++++) and amylase (++++) activity.

**Table 4:** Hydrolytic activity of *Trichoderma* isolates.

S. no.	*Trichoderma* isolates	Catalase	Amylase	Protease	Chitinase	Cellulase	Pectinase
1	*T. asperellum* APSI1	**+++**	**++++**	**−**	+++	+++	+++
2	*T. harzianum* APSI2	**++**	**+**	**−**	++++	++++	++++
3	*T. longibrachiatum* AZNF1	**++**	**++**	**−**	++	++	+
4	*T. harzianum* AZNF2	**++**	**++**	**−**	+++	+++	++
5	*T. atroviridae* UPFBK1	**+++**	**+**	**++**	+++	+++	+++
6	*T. asperellum* UPFBK2	**++**	**++**	**+++**	++++	++++	++++
7	*T. afroharzianum* UPFBK3	**++**	**++**	**++**	+++	++++	++++
8	*T. asperellum* UPFBK4	**++**	**++**	**−**	+++	+++	++++
9	*T. virens* ANFHR10	**++**	**+++**	**++**	++++	+++	+++
10	*T. longibrachiatum* ANFHS3	**+**	**++**	**−**	++	++	+
11	*T.cremeum* EGFBS1	**++++**	**++++**	**−**	+	+	+
12	*T. virens* CHFIB1	**++**	**+++**	**+++**	++++	+++	+++

Lytic level was graded as + (low), ++ (moderate), or +++ (high), ++++ (very high).

**Table 5:** Qualitative and quantitative growth promotion activity of *Trichoderma* isolates.

S. no.	*Trichoderma* isolates	Ammonia	IAA μg /ml	GA μg /ml	HCN	Psiderophore	P	K	Zn
1	*T. asperellum* APSI1	**+++**	12.86 ± 0.10^h^	10.34 ± 0.12^d^	**++**	**++++**	**++++**	**++++**	**++++**
2	*T. harzianum* APSI2	**++++**	14.89 ± 0.08^e^	8.98 ± 0.10^f^	**−**	**+**	**−**	**−**	**−**
3	*T. longibrachiatum* AZNF1	**+++**	11.76 ± 0.0.15^j^	7.95 ± 0.05^i^	**−**	**+**	**−**	**++**	**++**
4	*T. harzianum* AZNF2	**+++**	15.37 ± 0.10^d^	8.73 ± 0.10^g^	**−**	**−**	**++**	**+**	**+**
5	*T. atroviridae* UPFBK1	**++++**	14.11 ± 0.16^f^	10.67 ± 0.09^c^	**++**	**++**	**+++**	**++**	**++**
6	*T. asperellum* UPFBK2	**+++**	13.68 ± 0.16^g^	9.57 ± 0.04^e^	**−**	**++++**	**+++**	**++++**	**++++**
7	*T. afroharzianum* UPFBK3	**+**	19.29 ± 0.05^a^	11.87 ± 0.09^a^	**+++**	**++++**	**++++**	**++++**	**++++**
8	*T. asperellum* UPFBK4	**+++**	12.59 ± 0.09^i^	9.08 ± 0.10^f^	**++**	**++++**	**++++**	**++++**	**++++**
9	*T. virens* ANFHR10	**+**	18.96 ± 0.08^b^	10.98 ± 0.10^b^	**++++**	**+++**	**++++**	**+++**	**+++**
10	*T. longibrachiatum* ANFHS3	**++**	10.87 ± 0.11^k^	7.49 ± 0.06^j^	**−**	**++**	**++**	**++**	**++**
11	*T.cremeum* EGFBS1	**++**	14.95 ± 0.11^e^	8.56 ± 0.10^h^	**++**	**++**	**+**	**++**	**++**
12	*T. virens* CHFIB1	**+**	17.03 ± 0.062^c^	10.83 ± 0.08^b^	**++++**	**+++**	**+++**	**+++**	**+++**
	CD (0.05)		0.184	0.148					
	CV (%)		0.742	0.916					

In a column, means followed by a common letter(s) are not significantly different by LSD (*P* = 0.05). Production level was graded as + (low), ++ (moderate), or +++ (high), ++++ (very high). The amount of IAA and GA was calculated from the standard curve. y = 0.011x + 0.0057, R^2^ = 0.997.

*T. afroharzianum* UPFBK3 exhibited highest growth promotion activity was the strongest performer with high ammonia (+++), the highest IAA (19.25 μg/ mL), GA (11.87 19.25 μg/ mL) which were significantly greater than other isolates (LSD₀.₀₅: IAA = 0.184, GA = 0.148). This isolate also exhibited excellent solubilization of phosphorus (++++), potassium (++++), and zinc (++++). The second best isolate, *T. virens* ANFHR10 demonstrated high IAA (17.01–18.96 μg/ mL), GA (10.83–10.98 μg/mL), phosphorus (+++), potassium (+++), and zinc (+++) solubilization, along with superior siderophore (+++/++++) production. Conversely, *T. longibrachiatum* AZNF1 was the least effective, displaying the lowest IAA (10.87 μg/mL), GA (7.49 μgmL^−1^)), and moderate-to-low solubilization of phosphorus (++), potassium (++), and zinc (++). In conclusion, *T. afroharzianum* UPFBK3 emerged as the most effective strain among tested isolates, demonstrating both the highest growth-promotion activity and strong hydrolytic enzyme production.

### 3.5 Detection of antimicrobial spectrum of *Trichoderma* isolates

Among the *Trichoderma* isolates tested, *T. afroharzianum* UPFBK3 demonstrated the broadest and most consistent antimicrobial activity, with statistically significant inhibition (*p* < 0.05) across all pathogens, including *R. bataticola* (100%), *F. solani* (100%), *L. theobromae* (97.77%), *C. gleosporoides* (92.22%), *R. solani* (86.66%), and *C. lunata* (72.22%). Close behind was *T. asperellum* UPFBK4, which showed comparable results of 97.79% against *L. theobromae* and 72.21% against *C. lunata*. In contrast, *T. virens* CHFIB1 demonstrated the least broad-spectrum efficiency, with significantly lower inhibition rates of 60.65% against *C. lunata* and 85.81% against *C. gloeosporoides*. Moreover, all *Trichoderma* isolates achieved complete inhibition (100%) of *R. bataticola* and *F. solani* with significant variation among isolates for *R. solani*, *L. theobromae*, *C. gloeosporoides*, and *C. lunata* (p < 0.05, CD₀.₀₅: 0.02–0.03) (Table 6). These findings emphasize the superiority of *T. afroharzianum* UPFBK3, and *T. asperellum* UPFBK4, as potent biocontrol agents for managing multiple pathogens.

**Table 6:** Antimicrobial property of *Trichoderma* species.

S. no.	Isolates	Percent inhibition on mycelial growth					
		*Rhizoctonia solani*	*Rhizoctonia bataticola*	*Fusarium solani*	*Lasiodeplodia theobromae*	*Colletotrichum gleosporoides*	*Curvularia lunata*
1	*T. asperellum* APSI1	77.51 (8.80) ± 0.23^d^	100 ± 0.00	100 ± 0.00	92.01 (9.59) ± 0.37^ef^	93.58 (9.67) ± 0.34^b^	66.64 (8.16) ± 0.31^d^
2	*T. harzianum* APSI2	72.33 (8.51) ± 0.51^i^	100 ± 0.00	100 ± 0.00	91.72 (9.58) ± 0.11^fg^	93.09 (9.65) ± 0.21^c^	65.88 (8.12) ± 0.31^e^
3	*T. longibrachiatum* AZNF1	76.57 (8.75) ± 0.56^e^	100 ± 0.00	100 ± 0.00	93.34 (9.66) ± 0.49^d^	94.70 (9.73) ± 0.23^a^	71.18 (8.44) ± 0.09^c^
4	*T. harzianum* AZNF2	73.69 (8.58) ± 0.34^h^	100 ± 0.00	100 ± 0.00	91.37 (9.56) ± 0.14^g^	92.56 (9.62) ± 0.07^d^	63.45 (7.97) ± 0.13^f^
5	*T. atroviridae* UPFBK1	76.19 (8.73) ± 0.25 ^ef^	100 ± 0.00	100 ± 0.00	97.82 (9.89) ± 0.19^a^	92.21 (9.60) ± 0.01^e^	66.85 (8.18) ± 0.10^d^
6	*T. asperellum* UPFBK2	85.83 (9.27) ± 0.27^a^	100 ± 0.00	100 ± 0.00	97.78 (9.89) ± 0.01^a^	92.24 (9.60) ± 0.02^e^	71.67 (8.47) ± 0.36^b^
7	*T. afroharzianum* UPFBK3	86.35 (9.29) ± 0.57^a^	100 ± 0.00	100 ± 0.00	97.77 (9.89) ± 0.04^a^	92.25 (9.61) ± 0.04^e^	72.27 (8.50) ± 0.36^a^
8	*T. asperellum* UPFBK4	84.84 (9.21) ± 0.17^b^	100 ± 0.00	100 ± 0.00	97.79 (9.89) ± 0.04^a^	92.24 (9.60) ± 0.02^e^	72.21 (8.50) ± 0.15^a^
9	*T. virens* ANFHR10	75.74 (8.70) ± 0.21 ^fg^	100 ± 0.00	100 ± 0.00	96.70 (9.83) ± 0.13^b^	87.09 (9.33) ± 0.06^g^	63.43 (7.96) ± 0.47^f^
10	*T. longibrachiatum* ANFHS3	76.63 (8.75) ± 0.33 ^e^	100 ± 0.00	100 ± 0.00	95.76 (9.79) ± 0.32^c^	88.97 (9.43) ± 0.32^f^	62.25 (7.89) ± 0.19^g^
11	*T.cremeum* EGFBS1	75.46 (8.69) ± 0.24 ^g^	100 ± 0.00	100 ± 0.00	92.34 (9.61) ± 0.07^e^	87.16 (9.34) ± 0.06^g^	65.53 (8.10) ± 0.25^e^
12	*T. virens* CHFIB1	83.41 (9.13) ± 0.23^c^	100 ± 0.00	100 ± 0.00	96.88 (9.84) ± 0.35^b^	85.81 (9.26) ± 0.05^h^	60.65 (7.79) ± 0.10^h^
	CD (0.05)	0.03	NS	NS	0.02	0.02	0.03
	CV	0.23	–	–	0.13	0.09	0.20

Figures in the parentheses were square root transformed; in a column, means followed by a common letter(s) are not significantly different by LSD (*P* = 0.05).

### 3.6 Efficacy of *Trichoderma* species on the inhibition of BSR disease in oil palm seedlings

The evaluation of five best *Trichoderma* isolates and their combinations for their antagonistic potential against *Ganoderma* spp. revealed statistically significant variability (p < 0.05), with a critical difference of CD₀.₀₅: 0.25 for bole severity and 0.26 for foliar severity. In their effectiveness in controlling foliar and bole diseases (Figure 5 and Table 7). The combination of *Trichoderma* isolates (T_6_) demonstrated the highest BSR disease suppression, with a significant reduction in foliar severity and bole severity, corresponding to disease suppression rates of 50.97 and 61.94%, respectively. Among individual isolates, *T. afroharzianum* (T_4_) exhibited the second-highest reduction, achieving 49.92% reduction in foliar severity and 56.69% reduction in bole severity. In contrast, the control treatment (*G. ellipsoideum*) exhibited the highest severity for both foliar (99.56%) and bole (82.15%) infections, with no suppression of the disease. The *Trichoderma* consortium (T_6_) demonstrated 2.1% greater suppression of foliar disease, and 9.3% higher suppression of bole disease compared to *T. afroharzianum* (T_4_), reinforcing the superior efficacy of multi-strain interactions in controlling *Ganoderma*-induced disease in oil palm.

**Table 7:** In planta efficacy of *Trichoderma* species in suppressing BSR severity in oil palm seedling.

Treatment	Isolate	Foliar severity	Per cent disease suppression	Bole severity	Per cent disease suppression
T1	*T. asperellum* UPFBK4	65.82 (8.10) ± 7.06^b^	33.75	34.76 (5.89) ± 2.96^b^	47.39
T2	*T. longibrachiatum* AZNF1	55.55 (7.45) ± 5.57^c^	44.01	28.46 (5.33) ± 2.41^c^	53.69
T3	*T. virens* ANFHR10	52.16 (7.22) ± 2.86^cd^	47.40	27.49 (5.24) ± 3.06^cd^	54.66
T4	*T. afroharzianum* UPFBK3	49.65 (7.05) ± 1.64^d^	49.92	25.47 (5.04) ± 3.14^d^	56.69
T5	*T. atroviridae* UPFBK1	55.55 (7.45) ± 4.27^c^	44.01	27.74 (5.26) ± 1.96^cd^	54.41
T6	Combination^#^	48.59 (6.97) ± 3.97^d^	50.97	20.22 (4.48) ± 3.40^e^	61.94
T7	Control- *G. ellipsoideum*	99.56 (9.98) ± 0.75^a^	0.00	82.15 (9.06) ± 5.52^a^	0.00
	CD (0.05)	0.26	–	0.25	–
	CV	3.78	–	4.87	–

Figures in the parentheses were square root transformed; in a column, means followed by a common letter(s) are not significantly different by LSD (*P* = 0.05). ^#^Combination- *T. asperellum* UPFBK4 + *T. longibrachiatum* AZNF1 + *T. virens* ANFHR10 + *T. afroharzianum* (UPFBK3) + *T. atroviridae* UPFBK2.

### 3.7 Impact of *Trichoderma* on growth characteristics of oil palm seedling

A comparative analysis of growth parameters among different *Trichoderma* isolates and their effects on plant growth statistically significant differences between treatments (CD₀.₀₅: 1.06–1.69 (Table 8). The combination treatment (T_6_) showed the highest values across several parameters, including plant height (47.59 ± 2.52 cm), shoot fresh weight (15.83 ± 0.80 g), shoot dry weight (4.55 ± 0.26 g), and stem girth (8.60 ± 2.69 cm). This was followed closely by *T. afroharzianum* (T_4_), with a plant height of 46.54 ± 2.37 cm, shoot fresh weight of 13.37 ± 2.33 g, and root length of 24.10 ± 0.97 cm. In contrast, the control treatment with *G. ellipsoideum* (T_7_) exhibited the lowest values across all parameters, with plant height (5.61 ± 0.29 cm) and shoot fresh weight (0.60 ± 0.05 g), indicating a detrimental effect on plant growth. The control group (T_8_) showed relatively high growth metrics, but the combination treatment significantly outperformed it in most categories.

**Table 8:** In planta growth promotion activities of *Trichoderma* species in oil palm seedling.

Treatments	Isolate	Plant height (cm)	Shoot fresh weight (g)	Shoot dry weight	Root length (cm)	Root fresh weight (g)	Root dry weight (g)	Stem girth (cm)
T1	*T. asperellum* UPFBK4	40.452 ± 0.60^d^	11.28 ± 0.18^d^	2.18 ± 0.36^f^	20.38 ± 1.09^c^	4.35 ± 0.34^d^	1.00 ± 0.25^d^	7.58 ± 2.40^a^
T2	*T. longibrachiatum* AZNF1	43.106 ± 1.10^c^	12.21 ± 0.68^cd^	2.74 ± 0.18^e^	22.97 ± 3.07^b^	5.08 ± 0.39^c^	1.57 ± 0.57^c^	8.299 ± 1.64^a^
T3	*T. virens* ANFHR10	33.792 ± 0.78^e^	9.93 ± 0.36^e^	1.87 ± 0.64^f^	19.05 ± 1.31^cd^	6.76 ± 0.86 ^b^	2.40 ± 0.68^b^	8.182 ± 1.25^a^
T4	*T. afroharzianum* UPFBK3	46.539 ± 2.37 ^a^	13.37 ± 2.33^b^	3.44 ± 0.37^d^	24.10 ± 0.97^b^	4.01 ± 0.08^de^	1.03 ± 0.54^d^	8.39 ± 1.03^a^
T5	*T. atroviridae* UPFBK1	35.372 ± 2.69^e^	9.83 ± 1.28^e^	1.01 ± 0.43^g^	18.78 ± 1.65^d^	3.60 ± 0.52^e^	1.06 ± 0.19^d^	8.244 ± 1.98^a^
T6	Combination^#^	47.586 ± 2.52^a^	15.83 ± 0.80^a^	4.55 ± 0.26^c^	24.33 ± 1.57^b^	4.98 ± 0.61^c^	1.51 ± 0.58^c^	8.604 ± 2.69^a^
T7	Control-*G. ellipsoideum*	5.613 ± 0.29^f^	0.60 ± 0.05^f^	8.89 ± 0.73^b^	2.34 ± 0.16^e^	2.60 ± 0.45^f^	0.91 ± 0.08^d^	3.634 ± 0.82^b^
T8	Control-Untreated	44.768 ± 2.35^b^	12.47 ± 1.48^bc^	11.79 ± 0.45^a^	30.36 ± 1.25^a^	9.39 ± 0.89 ^a^	2.974 ± 0.55^a^	8.49 ± 1.7^a^
	CD (0.05)	1.69	1.06	0.40	1.47	0.53	0.43	1.62
	CV	4.87	11.07	9.75	8.10	11.74	30.57	23.62

In a column, means followed by a common letter(s) are not significantly different by LSD (*P* = 0.05). Combination^#-^ *T. asperellum* (UPFBK4) + *T. longibrachiatum* (AZNF1) + *T. virens* (ANFHR10) + *T. afroharzianum* (UPFBK3) + *T. atroviridae* (UPFBK2).

### 3.8 SEM analysis

Distinct morphological features were observed in *G. ellipsoideum* and *T. afroharzianum* UPFBK3. *G. ellipsoideum* exhibited tubular hyphae with clearly defined clamp connections, indicative of basidiomycetous fungi (Figure 4A). In contrast, *T. afroharzianum* UPFBK3 showed highly branched hyphae bearing flask-shaped phialides and numerous ellipsoidal to subglobose conidia (Figures 4B,C). Direct interaction between the two fungi revealed pronounced hyphal damage in *G. ellipsoideum* upon contact with *T. afroharzianum* UPFBK3. The damaged hyphae exhibited surface degradation and structural collapse, suggesting antagonistic activity likely associated with mycoparasitism (Figure 4D). In addition, adherence of *T. afroharzianum* UPFBK3 to the plant root surface was evident, demonstrating its colonization potential and ecological competence in the rhizosphere.

## 4 Discussion

**Figure 6:**
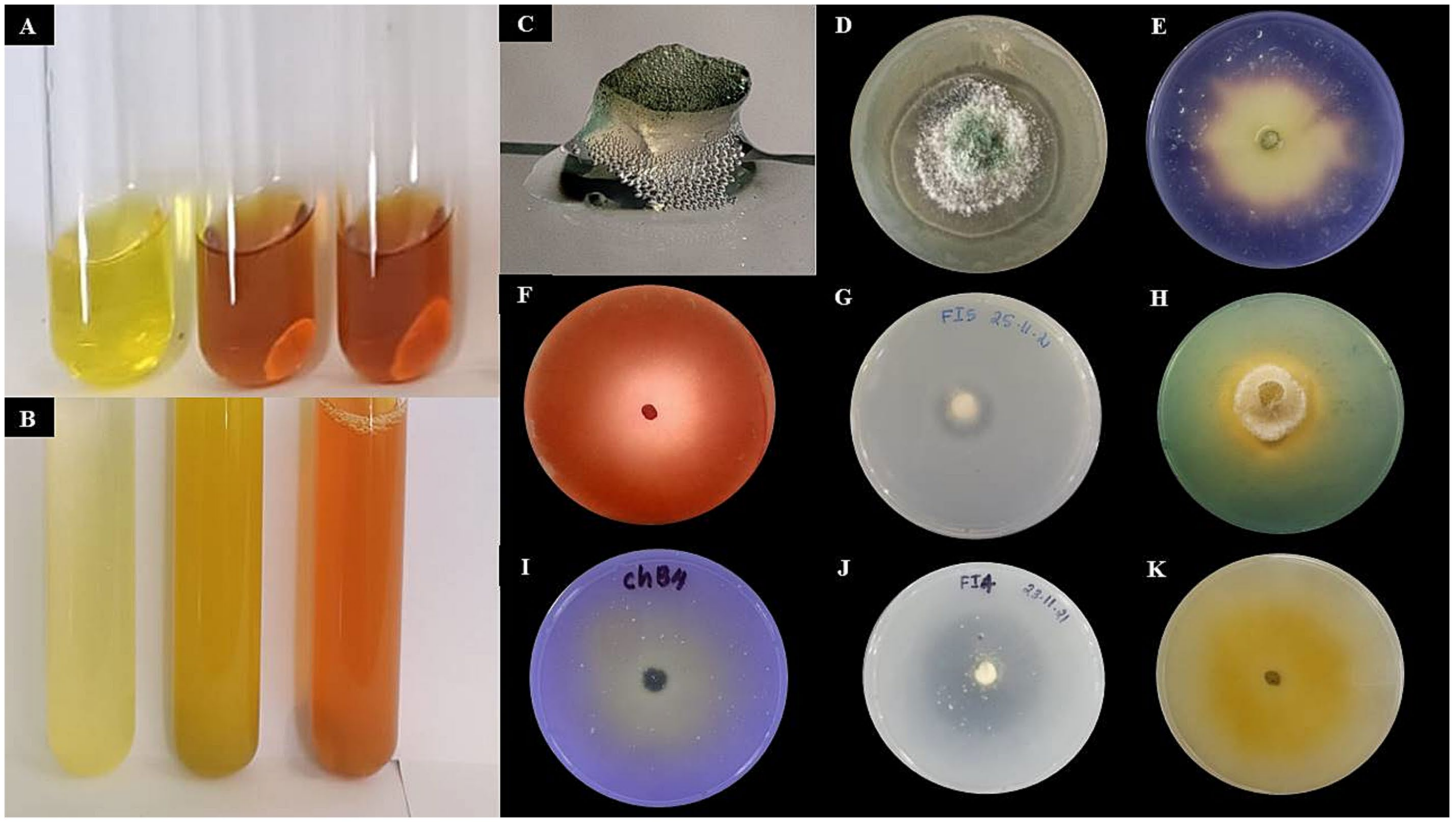
Qualitative assessment of hydrolytic enzymes and plant growth-promoting traits of *Trichoderma afroharzianum*. **(A)** Indole Acetic Acid (IAA) production indicated by red color development. **(B)** Ammonia production confirmed by brown color formation. **(C)** Catalase test signed by bubbling with H₂O₂. **(D)** Chitinase production indicted by halo around the fungal colony. **(E)** *α*-Amylase production denoted by clear zones after iodine staining indicate starch hydrolysis. **(F)** Cellulase production revealed by Congo red staining reveals clear zones, indicating cellulose degradation. **(G)** Protease production showed by transparent halo on skim milk agar. **(H)** Siderophore production indicated by yellow halos on CAS medium. **(I)** Phosphate solubilization confirmed by clear zones on NBRIP medium. **(J)** Potassium solubilization signed by halo formation on Aleksandrov medium.

Trichoderma is widely utilized as a biocontrol agent featured in more than 50% of commercial formulations for managing fungal, viral, and nematode-induced plant diseases (51–55). Despite the commercial success of species like *T. viride* and *T. harzianum* (56), the broader genus *Trichoderma*, which includes over 300 identified species (57), remains largely underutilized. This limited use highlights the untapped biocontrol potential of *Trichoderma* from diverse agroecological zones, where region-specific isolates present promising, locally adapted, and climate-resilient alternatives for more sustainable and broader plant disease management (58). Hence, the current study examines 50 *Trichoderma* isolates from diverse agro-ecological contexts, identifying 12 with promising antibiosis and growth promotion, and selecting the top 5 for *in planta* bioefficacy testing against *Ganoderma*. This study also morpho-molecularly characterized seven different *Trichoderma* species, including *T. afroharzianum*, *T. atroviride*, and *T. cremeum*, which to our knowledge, have not been previously reported in association with *Ganoderma*-infected oil palm systems. The consistent high performance of *T. afroharzianum* UPFBK3, *T. atrovirodae* UPFBK1, and *T. asperellum* UPFBK4 across dual culture, inverted plate, and cell filtrate assays underscores their robust and stable antagonistic potential against *Ganoderma*, making them strong candidates for development as effective biocontrol agents in sustainable disease management strategies. This antagonistic role of *Trichoderma* spp. is attributable to their unique ability to synthesize antibiotics, secondary metabolites (59), mycoparasitism (52) and lytic enzymes (60). The superior antagonistic ability *T. afroharzianum* UPFBK3 is attributed to its synthesis of bioactive compounds including synthesize secondary metabolites like spathulenol, triacetin, and aspartame, along with VOCs such as ethanol, hydroperoxide, 1-methylhexyl, and 1-octen-3-one (61). The demonstrated effectiveness of *T. afroharzianum* strains—B3R12 against onion white rot (62), TRI07 against *Alternaria alternata* (61), and T14 against wheat crown rot and Fusarium head blight (63), supports the generalizability of *Trichoderma* strains as a versatile biocontrol agent. Similarly, *T. asperellum* UPFBK4, which ranked second in our study, has consistently shown potent inhibition of *G. boninense* and *G. lucidum* (13, 64, 65). This reinforces the strain’s potential for effective biocontrol, emphasizing the robustness and reliability of *Trichoderma* species as effective biocontrol agents across a range of pathogenic threats (Figure 6).

**Figure 7:**
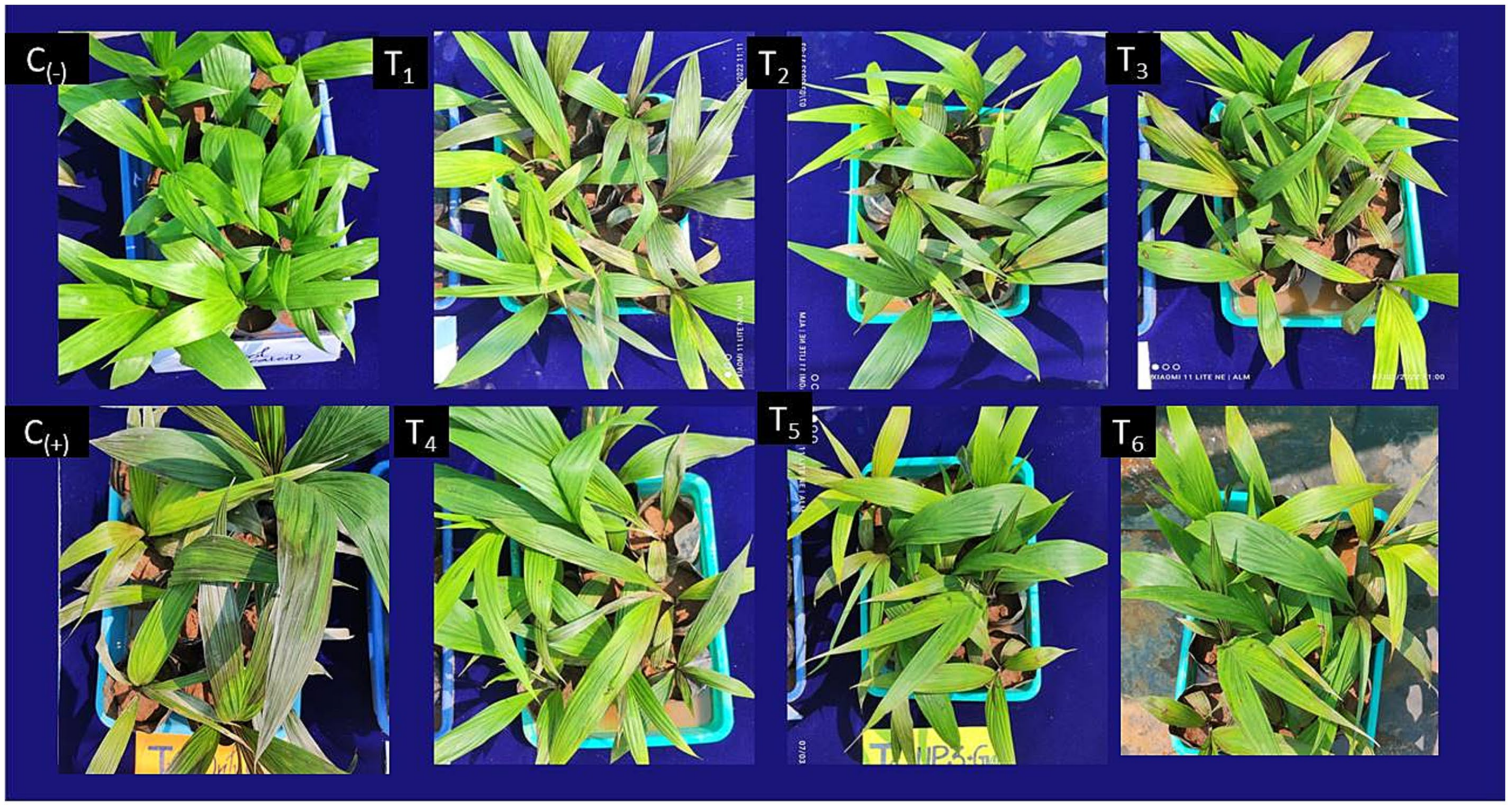
Effect of *Trichoderma* spp. on basal stem rot (BSR) disease suppression in oil palm seedlings. (C₋) Negative control: Healthy seedlings without *Ganoderma* inoculation; (C₊) Positive control: Seedlings inoculated with *Ganoderma* sp. without *Trichoderma* treatment; T₁: *Trichoderma asperellum*UPFBK4; T_2_: *Trichoderma longibrachiatum* AZNF1; T_3_: *Trichoderma virens*ANFHR10; T_5_: *Trichoderma afroharzianum* UPFBK3; (T₅) *Trichoderma atroviride* UPFBK1; T₆: Combination treatment.

The plant-microbe connection gives the host plants a selective advantage when they are under distress (66, 67). Hence, this study investigates the biochemical mechanisms underlying the hydrolytic and growth-promoting activities of 12 potent *Trichoderma* isolates, reaffirming *T. afroharzianum* as the most effective due to its superior growth promotion and hydrolytic enzyme production. *Trichoderma* suppresses *Ganoderma* through oxidative enzymes like catalase, which mitigate oxidative stress, and hydrolytic enzymes that break down fungal cell walls to access nutrients and facilitate root colonization, ultimately enhancing plant association and resistance (68). In addition to these enzymatic actions, certain hydrolytic enzymes act as elicitors that trigger the release of Damage-Associated Molecular Pattern Molecules (DAMPs), which are endogenous plant signals released upon cellular damage (69). We further speculate that *Trichoderma*’s hyperparasitism against *Ganoderma* is strongly enhanced by the combined action of these lytic enzymes and the production of antibiotic metabolites, contributing to a potent antibiosis effect (62). The variability in production of secondary metabolites and hydrolytic enzymes could indeed explain the differences in performance observed among the *Trichoderma* isolates. Additionally, every strain has different genetic potential for parasitism; the nature of the pathogen and the cell wall of the pathogen play a critical role in the mycoparasitisms. The other factors, such as growth rate, isolate-specific physiological traits, and environmental interactions may have influenced the results (Figure 7).

The growth-promoting activity of *Trichoderma* stems from its capacity to produce siderophores and solubilize phosphorus, potassium, and zinc, which increases the availability of soluble soil nutrients and thereby benefiting the host plant by promoting growth and enhancing stress tolerance (70). Particularly, siderophore production enhances iron uptake in iron-deficient conditions (71, 72), while the availability of phosphorus is increased through the generation of organic acids like succinic acid, malic acid, and oxalic acid (73), and nitrogen is made available by hydrolyzing urea to produce ammonia (74). Further, numerous strains of *Trichoderma* release growth regulators such as indole-3-acetic acid (IAA) and gibberellic acid (GA), which alter plant metabolism, promote growth, and enhance stress resilience (50).

Our study identified *T. afroharzianum* UPFBK3, *T. asperellum* APSI1 & UPFBK4, and *T. virens* ANFHR10 & CHFIB1 as promising growth promoters, supporting previous findings. For instance, *T. virens* and *T. atroviride* were reported to produce IAA-related indoles, enhancing the fresh weight of tomato roots and shoots (75) and promoting lateral root formation in *Arabidopsis* (76). Additionally, Qi and Zhao (77) found *T. asperellum* to be the most efficient *Trichoderma* species for siderophore production under salt stress, effectively alleviating iron deficiency in plants. These findings align with our results; reinforcing the potential of these *Trichoderma* strains as effective growth promoters.The *in vitro* findings are insufficient to fully ascertain the possibility of *Trichoderma* spp. as effective bio-control agents in field conditions. Factors such as soil microbiota, environmental conditions (e.g., temperature, humidity), and host plant physiology can significantly impact their efficacy. Moreover, dual culture and inverted plate assays do not account for the complex interactions in the rhizosphere, where competition for resources and microbial dynamics play a critical role in biocontrol success. Therefore, integrating both *in vitro* and *in vivo* studies is essential for a comprehensive evaluation of their biocontrol capabilities (78). The present in planta study demonstrated that the *Trichoderma* consortium—comprising five most potent and compatible isolates (*T. afroharzianum*, *T. virens*, *T. asperellum*, *T. longibrachiatum* and *T. atroviride*)—resulted in greater suppression of disease severity and enhanced plant growth promotion. The thermotolerant *T. longibrachiatum* strain, isolated from the rhizosphere of *Prosopis cineraria* in Rajasthan’s > 50°C arid zone showed strong antagonism against *Ganoderma lucidum* (19). This enhanced efficacy is attributed to the broader spectrum of antagonistic activities offered by the consortium, where synergistic interactions among isolates with complementary strengths—such as mycoparasitism and extracellular enzyme production—led to cumulative effects that surpassed those of individual strains (70). This synergy is further supported by significant preventive effects against *Ganoderma*-induced BSR in oil palm (14), along with evidence that *Trichoderma* consortia enhance plant resistance and growth (79), improve nutrient uptake and biomass as seen with *T. viride*, *T. virens*, and *T. harzianum* in chickpeas (80), and effectively control *Rhizoctonia solani* through *T. harzianum* mixtures (81). Seedlings exhibited enhanced disease suppression and increased fresh and dry shoot and root weights, consistent with our in vitro findings where *Trichoderma afroharzianum* emerged as the most effective isolate, demonstrating superior antagonistic activity, hydrolytic enzyme production, growth promotion, faster growth rate, and a broader antimicrobial spectrum. These findings align with previous studies across diverse host–pathogen systems (61–63) and reinforce the well-documented growth-promoting and biocontrol potential of *Trichoderma* spp. in various crops including wheat (82), maize (70), cotton (83), legumes (84), and tree crops like cocoa and *Pinus radiata* (85, 86). While this study highlights the potential of region-specific *Trichoderma* isolates, particularly *T. afroharzianum*, further *in vivo* research is needed to assess their field efficacy (78). Additionally, more work is required to understand *Trichoderma’s* impact on resistance mechanisms, including systemic acquired resistance (SAR). Further studies are required to understand the factors contributing to performance variability, including environmental and biochemical influences on efficacy. This will be crucial for optimizing *Trichoderma* in sustainable, climate-resilient disease management strategies.

## 5 Conclusion

Our findings suggest that selected *Trichoderma* strains, particularly *T. afroharzianum*, possess strong biocontrol potential against *Ganoderma* spp. under laboratory and greenhouse conditions. The results indicate promising disease suppression efficacy and growth enhancement in oil palm seedlings. However, these outcomes are limited to controlled environments, and further multi-location field evaluations are required to confirm their effectiveness under diverse plantation conditions. Overall, this study provides foundational evidence supporting the use of ecologically adapted *Trichoderma* strains as components of an integrated, climate-resilient disease management strategy for oil palm.

## Data Availability

The datasets presented in this study can be found in online repositories. The names of the repository/repositories and accession number(s) can be found in the article/Supplementary material.

## Footnotes

^1^ https://oilseeds.dac.gov.in/

^2^ https://nmeo.dac.gov.in/
