# Supplementary material for: Bioprospecting and mechanistic insights of Trichoderma spp. for suppression of Ganoderma-induced basal stem rot in oil palm
Source: Front Nutr. 2025 Jul 10;12:1582047. doi: 10.3389/fnut.2025.1582047 (PMC12287078; doi:10.3389/fnut.2025.1582047)
Supplement: Supplementary file 1 [file Table_2.docx]

**Supplementary Table 1: Bioprospecting of Trichoderma isolates across different agroecological zones**

| **Sl.No** | **State** | **Isolate Designation** | **Source** | **Location and date of collection** | **Mean**± **SD** **PIRG** |
| --- | --- | --- | --- | --- | --- |
| 1 | Andaman & Nicobar (11) | ANFHS2 | Rhizosphere of healthy palm | Port Blair (7.3.2021) | 96±0.50 |
| 2 |  | ANFH3 | Rhizosphere of healthy palm | Port Blair (7.3.2021) | 94.18±0.79 |
| 3 |  | ANFI 4 | Roots of infected palm | Port Blair (7.3.2021) | 65.02±1 |
| 4 |  | ANFIB5 | Brackets of infected palm | Port Blair (7.3.2021) | 62±0 |
| 5 |  | ANFI6 | Roots of infected palm | Port Blair (7.3.2021) | 63±1.32 |
| 6 |  | ANFI 7 | Roots of infected palm | Port Blair (7.3.2021) | 91±1 |
| 7 |  | ANFI 8 | Roots of infected palm | Port Blair (7.3.2021) | 78.02±1.78 |
| 8 |  | ANFIB9 | Brackets of infected palm | Port Blair (7.3.2021) | 61±1 |
| 9 |  | ANFH10 | Roots of healthy palm | Port Blair (7.3.2021) | 96±0 |
| 10 |  | ANF.i11 | Rhizosphere of infected palm | Port Blair (7.3.2021) | 66±0 |
| 11 | Andhra Pradesh | KPFH1 | Rhizosphere of infected palm | Kuchumpudi (29.12.20) | 69.23±0.23 |
| 12 |  | ChFIB1 | Brackets of infected palm | Chintalapudi (27.04.21) | 92±0 |
| 13 |  | ChFB2 | Brackets of infected palm | Chintalapudi(27.04.21) | 86±1.50 |
| 14 |  | ChFB 3 | Brackets of infected palm | Chintalapudi (27.04.21) | 75±0 |
| 15 |  | ChFB4 | Brackets of infected palm | Chintalapudi (27.04.21) | 80±1 |
| 16 |  | EGFB1 | Brackets of infected palm | Rajahmundry (27.4.21) | 88±1.06 |
| 17 |  | EGFB2 | Brackets of infected palm | Rajahmundry (27.4.21) | 85±0.24 |
| 18 |  | EGFB3 | Brackets of infected palm | Rajahmundry (27.4.21) | 94±0 |
| 19 |  | EGFB4 | Brackets of infected palm | Rajahmundry (27.4.21) | 79.40±1.26 |
| 20 |  | EGFYM | FYM Clods | Rajahmundry(1.11.21) | 55±1 |
| 21 |  | NZFIB1 | Brackets of infected palm | Nuzividu (10.7.21) | 43.33±0.58 |
| 22 |  | AGFIB1 | Brackets of infected palm | Ayyapparajagudeum (27.04.21) | 47±0.95 |
| 23 |  | AGFIB2 | Brackets of infected palm | Ayyapparajagudeum (27.04.21) | 56±0.04 |
| 24 |  | ChLFIB1 | Brackets of infected palm | Chalachintapudi (2.12.21) | 45±1 |
| 25 |  | CDFIB1 | Brackets of infected palm | CD Block IIOPR (3.12.21) | 57±1 |
| 26 |  | SNFIBI | Brackets of infected palm | Sitanagaram (27.04.21) | 43.33±0.58 |
| 27 | Uttar Pradesh  (12) | UPFBK1 | Bark of forest trees | Mau (29.11.21) | 95.67±0.58 |
| 28 |  | UPFBK2 | Bark of forest trees | Mau (29.11.21) | 97.77±1.57 |
| 29 |  | UPFBK3 | Bark of forest trees | Mau (29.11.21) | 98.77±0.11 |
| 30 |  | UPFBK4 | Bark of forest trees | Mau (29.11.21) | 97.77±1.86 |
| 31 |  | UPFBK5 | Bark of forest trees | Mau (29.11.21) | 60±0 |
| 32 |  | UPFBK6 | Bark of forest trees | Mau (29.11.21) | 55±1 |
| 33 |  | UPFBK7 | Bark of forest trees | Mau (29.11.21) | 60±0.91 |
| 34 |  | UPFBK8 | Bark of forest trees | Mau (29.11.21) | 75±1 |
| 35 |  | UPFBK9 | Bark of forest trees | Mau (29.11.21) | 43.30±0.60 |
| 36 |  | UPFBK10 | Bark of forest trees | Mau (29.11.21) | 46±1 |
| 37 |  | UPFBK11 | Brackets of infected tree | Mau (29.11.21) | 60±0 |
| 38 |  | UPFBK12 | Brackets | Mau (29.11.21) | 57±0.87 |
| 39 | Telengana (5) | TSFKMS1 | Soil | Khammam (5.10.2020) | 43.33±0.58 |
| 40 |  | TSFAPBK2 | Brackets of infected tree | Asraopeta (5.10.2020) | 72.45±0.78 |
| 41 |  | TSFMPBK3 | Brackets of infected tree | Medisettivaripalem (20.11.2023) | 65.23±0.92 |
| 42 |  | TSFMPBK4 | Brackets of infected tree | Medisettivaripalem (20.11.2023) | 65.24±1 |
| 43 |  | TSFMPBK5 | Brackets of infected tree | Medisettivaripalem (20.11.2023) | 54.35±0.56 |
| 44 | Kerala (6) | KLFPR1 | Roots of infected tree | IIOPR, RC Palode (12.12.21) | 78.50±0.71 |
| 45 |  | KLFPR2 | Roots of infected tree | IIOPR, RC Palode(12.12.21) | 67.77±0.68 |
| 46 |  | KLFPR3 | Roots of infected tree | IIOPR, RC Palode(12.12.21) | 65.99±0.86 |
| 47 |  | KLFPBK1 | Brackets of infected tree | IIOPR, RC Palode(12.12.21) | 78.95±1.14 |
| 48 |  | KLFPBK2 | Brackets of infected tree | IIOPR, RC Palode(12.12.21) | 77.77±1.33 |
| 49 |  | KLFPBK3 | Brackets of infected tree | IIOPR, RC Palode(12.12.21) | 69.99±1.26 |
| 50 |  | KLFPBK4 | Brackets of infected tree | IIOPR, RC Palode(12.12.21) | 69.99±0.81 |

**PIRG**-Percentage Inhibition of Radial Growth; **SD –** Standard Deviation

**Supplementary Table 2: Morphological characterisation of Trichoderma isolates**

| **Sl.No** | **Species** | **Colony Features** | **Odor** | **Pigmentation**  **Front/reverse** | **Conidiophore arrangement** | **Phialides** | **Conidia** |
| --- | --- | --- | --- | --- | --- | --- | --- |
| 1 | *T. longibrachiatum* | Dense, limited aerial mycelium, white to green colonies | Musty/Earthy | Dark green /yellow | Highly branched, Long and slender primary and  paired secondary branches | Elongated, flask-shaped arises acute angledirectly from secondary branches, typically not in whorls | Ellipsoidal, smooth-walled |
| **2** | *T. harzianum* | Dense, smoothgreenish tufts on white mycelium with regular margin | Sweet/ earthy | Dark green to olive-green/ yellow | Short and more regular branching than *afroharzianum* | Short, flask shaped, densely arranged in whorls | small, globose-subglobose smooth-walled conidia |
| **3** | *T. atroviridae* | Dense, compact and smooth colonies that appear white initially and turn dark green with sporulation lacks clear concentric growth rings. | Earthy/ slightly sweet odor | Dark green / yellow-brown | Short, compact, irregularly branched | Short flask-shaped, closely packed, with swollen bases and tapering tips. | Globose-subglobose smaller than *T.asperellum* |
| **4** | *T. asperellum* | velvety to powdery No aerial mycelium, loose or fluffy white mycelium turning green with sporulation.  5 concentric rings of dense conidial production | Weak to mild earthy | Light-dark green/colourless. | Long, with primary branch perpendicular to main axis terminating in 2 or more phialides | Flask-shaped held at a sharp angle, with a slightly enlarged in the middle and narrower and elongated neck. | Oval-ellipsoidal |
| **5** | *T. afroharzianum* | Fluffy/slightly woolier white mycelium that turns green as conidia are produced. More irregular margins | Mild earthy odor. | Darker green than harzianum/ colourless | Branched with slightly irregular branching | Flask-shaped, slightly elongated than harzianum, swollen at the base, arising singly or in groups. | Ellipsoidal to subglobose smooth-walled |
| **6** | *T. virens* | Fast-growing, initially white colonies turning green with conidiation. | Strong earthy or musty odor. | Pale green/ yellow or brown pigmentation. | Long, straight or flexuous, with short lateral branches | Flask-shaped, short, and arranged in whorls or in dense clusters. | Round to subglobose, smooth-walled |
| **7** | *T. cremeum* | Slow to moderate growth with a cream-colored to light yellow mycelium. | Mild, neutral odor. | Cream to pale green/cream-colored. | Simple to sparsely branched, shorter compared to other species. | Elongated, cylindrical to flask-shaped, arising directly from conidiophores with minimal branching. | Round to ovoid, smooth-walled, initially hyaline and turning pale green, |

**Supplementary Table 3: Details on the ITS1&4 consensus sequences of Trichoderma isolates**

| **Sl.**  **No** | ***Trichoderma* isolate** | **Accession**  **Number^*^** | **Consensus sequence** |
| --- | --- | --- | --- |
| **1** | *Trichoderma*  *afroharzianum* strain UPFBK-3 | OR244373 | GGAGGGATCATTACCGAGTTTACAACTCCCAAACCCCATGTGAACGTTACCAAACTGTTGCCTCGGCGGGATCTCTGCCCCGGGTGCGTCGCAGCCCCGGACCAAGGCGCCCGCCGGAGGACCAACCAAAACTCTTATTGTATACCCCCTCGCGGGTTTTTTTATAATCTGAGCCTTCTCGGCGCCTCTCGTAGGCGTTTCGAAAATGAATCAAAACTTTCAACAACGGATCTCTTGGTTCTGGCATCGATGAAGAACGCAGCGAAATGCGATAAGTAATGTGAATTGCAGAATTCAGTGAATCATCGAATCTTTGAACGCACATTGCGCCCGCCAGTATTCTGGCGGGCATGCCTGTCCGAGCGTCATTTCAACCCTCGAACCCCTCCGGGGGGTCGGCGTTGGGGATCGGCCCTGCCTTGGCGGTGGCCGTCTCCGAAATACAGTGGCGGTCTCGCCGCAGCCTCTCCTGCGCAGTAGTTTGCACACTCGCATCGGGAGCGCGGCGCGTCCACAGCCGTTAAACACCCAACTTCTGAAATGTTGACCTCGGATCAGGTAGGAATACCCGCTGAACTTAAGCAT |
| **2** | *Trichoderma virens* strain CHFIB1 | OR244374 | GGAGGGATCATTACCGAGTTTACAACTCCCAAACCCAATGTGAACGTTACCAAACTGTTGCCTCGGCGGGATCTCTGCCCCGGGTGCGTCGCAGCCCCGGACCAAGGCGCCCGCCGGAGGACCAACCAAAACTCTTATTGTATACCCCCTCGCGGGTTTTTTACTATCTGAGCCATCTCGGCGCCCCTCGTGGGCGTTTCGAAAATGAATCAAAACTTTCAACAACGGATCTCTTGGTTCTGGCATCGATGAAGAACGCAGCGAAATGCGATAAGTAATGTGAATTGCAGAATTCAGTGAATCATCGAATCTTTGAACGCACATTGCGCCCGCCAGTATTCTGGCGGG  CATGCCTGTCCGAGCGTCATTTCAACCCTCGAACCCCTCCGGGGGGTCGGCGTTGGGGATCGGCCCTTTACGGGGCCGGCCCCGAAATACAGTGGCGGTCTCGCCGCAGCCTCTCCTGCGCAGTAGTTTGCACACTCGCATCGGGAGCGCGGCGCGTCCACAGCCGTTAAACACCCCAAACTTCTGAAAT |
| **3** | *Trichoderma longibrachiatum* strain ANFHS3 | OR244375 | ATTACCGAGTTTACAACTCCCAAACCCCAATGTGAACGTTACCAATCTGTTGCCTCGGCGGGATTCTCTTGCCCCGGGCGCGTCGCAGCCCCGGATCCCATGGCGCCCGCCGGAGGACCAACTCCAAACTCTTTTTTTCTCTCCGTCGCGGCTCCCGTCGCGGCTCTGTTTTATTTTTGCTCTGAGCCTTTCTCGGCGACCCTAGCGGGCGTCTCGAAAATGAATCAAAACTTTCAACAACGGATCTCTTGGTTCTGGCATCGATGAAGAACGCAGCGAAATGCGATAAGTAATGTGAATTGCAGAATTCAGTGAATCATCGAATCTTTGAACGCACATTGCGCCCGCCAGTATTCTGGCGGGCATGCCTGTCCGAGCGTCATTTCAACCCTCGAACCCCTCCGGGGGGTCGGCGTT  GGGGATCGGCCCCTCACCGGGCCGCCCCCGAAATACAGTGGCGGTCTCGCCGCAGCCTCTCCTGCGCAGTAGTTTGCACACTCGCACCGGGAGCGCGGCGCGGCCACAGCCGTAAAACACCCCAAACTTCTGAAATGGTTGA |
| **4** | *Trichoderma virens* strain ANFHR10 | OR244376 | TGGTGAACCAGCGGAGGGATCATTACCGAGTTTACAACTCCCAAACCCAATGTGAACGTTACCAAACTGTTGCCTCGGCGGGATCTCTGCCCCGGGTGCGTCGCAGCCCCGGACCAAGGCGCCCGCCGGAGGACCAACCAAAACTCTTATTGTATACCCCCTCGCGGGTTTTTTACTATCTGAGCCATCTCGGCGCCCCTCGTGGGCGTTTCGAAAATGAATCAAAACTTTCAACAACGGATCTCTTGGTTCTGGCATCGATGAAGAACGCAGCGAAATGCGATAAGTAATGTGAATTGCAGAATTCAGTGAATCATCGAATCTTTGAACGCACATTGCGCCCGCCAGTATTCTGGCGGGCATGCCTGTCCGAGCGTCATTTCAACCCTCGAACCCCTCCGGGGGGTCGGCGTTGGGGATCGGCCCTTTACGGGGCCGGCCCCGAAATACAGTGGCGGTCTCGCCGCAGCCTCTCCTGCGCAGTAGTTTGCACACTCGCATCGGGAGCGCGGCGCGTCCACAGCCGTTAAACACCCCAAACTTCTGAAATGTTGACCTCGGATCAGGTAGGAATACCCGCTGAACTTAAGCAT |
| **5** | *Trichoderma asperellum* strain UPFBK2 | OR244377 | GCTGTTATGTTAGAAAAGTCGTGATCAACAGTCTTCTGCTGGTGGGGACAGCGGAGGTGATCATCAGCCGAGCTTTCAACTAACAAACGCAATGTGAACGTTACCAAACTGTTGCCTCGGCGGGGTCACGCCCCGGGTGCGTCGCAGCCCCGGAACCAGGCGCCCGCCGGAGGAACCAACCAAACTCTTTCTGTAGTCCCCTCGCGGACGTATTTCTTTACAGCTCTGAGCAAAAATTCAAAATGAATCAAAACTTTCAACAACGGATCTCTTGGTTCTGGCATCGATGAAGAACGCAGCGAAATGCGATAAGTAATGTGAATTGCAGAATTCAGTGAATCATCGAATCTTTGAACGCACATTGCGCCCGCCAGTATTCTGGCGGGCATGCCTGTCCGAGCGTCATTTCAACCCTCGAACCCCTCCGGGGGATCGGCGTTGGGGATCGGGACCCCTCACACGGGTGCCGGCCCCTAAATACAGTGGCGGTCTCGCCGCAGCCTCTCCTGCGCAGTAGTTTGCACAACTCGCACCGGGAGCGCGGCGCGTCCACGTCCGTAAAACACCCAACTTTCTGAAATGTGACCTCGATCAGGTAGAATGCCCCGGT |
| **6** | *Trichoderma asperellum* strain UPFBK4 | OR244378 | TGGGAAGGGTTTGGAAGTCAAAGTTGGAACAAGGTATCTGTTGGTGGACCTGCGGAGGGATCACTGCCGAGTTTATAACTCCCAAACCCAATGTGAACGTTACCAAACTGTTGCCTCGGCGGGGTCACGCCCCGGGTGCGTCGCAGCCCCGGAACCAGGCGCCCGCCGGAGGAACCAACCAAACTCTTTCTGTAGTCCCCTCGCGGACGTATTTCTTTACAGCTCTGAGCAAAAATTCAAAATGAATCAAAACTTTCAACAACGGATCTCTTGGTTCTGGCATCGATGAAGAACGCAGCGAAATGCGATAAGTAATGTGAATTGCAGAATTCAGTGAATCATCGAATCTTTGAACGCACATTGCGCCCGCCAGTATTCTGGCGGGCATGCCTGTCCGAGCGTCATTTCAACCCTCG  AACCCCTCCGGGGGATCGGCGTTGGGGATCGGGACCCCTCACACGGGTGCCGGCCCCTAAATACAGTGGCGGTCTCGCCGCAGCCTCTCCTGCGCAGTAGTTTGCACAACTCGCACCGGGAGCGCGGCGCGTCCACGTCCGTAAAACACCCAACTTTCTGAAATGTGACCTCGGATCAGGAGTAATACACGG |
| **7** | *Trichoderma*  atroviride strain UPFBK1 | OR244379 | GTAAAAGTCGTAACAAGGTCTCCGTTGGTGAACCAGCGGAGGGATCATTACCGAGTTTACAACTCCCAAACCCAATGTGAACCATACCAAACTGTTGCCTCGGCGGGGTCACGCCCCGGGTGCGTCGCAGCCCCGGAACCAGGCGCCCGCCGGAGGGACCAACCAAACTCTTTCTGTAGTCCCCTCGCGGACGTTATTTCTTACAGCTCTGAGCAAAAATTCAAAATGAATCAAAACTTTCAACAACGGATCTCTTGGTTCTGGCATCGATGAAGAACGCAGCGAAATGCGATAAGTAATGTGAATTGCAGAATTCAGTGAATCATCGAATCTTTGAACGCACATTGCGCCCGCCAGTATTCTGGCGGGCATGCCTGTCCGAGCGTCATTTCAACCCTCGAACCCCTCCGGGGGTCCGGCGTTGGGGATCGGGAACCCCTAAGACGGGATCCCGGCCCCGAAATACAGTGGCGGTCTCGCCGCAGCCTCTCATGCGCAGTAGTTTGCACAACTCGCACCGGGAGCGCGGCGCGTCCACGTCCGTAAAACACCCAACTTCTGAAATGTTGACCTCGGATCAGGTAGGAATACCCGCTGAACTTAAGCATATCAATAAGCGGAGG |
| **8** | *Trichoderma cremeum* EGFBS | OR244380 | ATCATTACCGAGTTTACAACTCCCAAACCCAATGTGAACGTTACCAAACTGTTGCCTCGGCGGGATCTCTGCCCCGGGCGCGTCGCAGCCCCGGACCAAGGCGCCCGCCGGAGGAAAAAACAACCAAAACTCTTTTTGTATACCCCCTCGCGGGTTTTTTACTTCTGAGAACTTCTCGGCGCCCCTTTGCGGGCGTTTCGAAAATGAATCAAAACTTTCAACAACGGATCTCTTGGTTCTGGCATCGATGAAGAACGCAGCGAAATGCGATAAGTAATGTGAATTGCAGAATTCAGTGAATCATCGAATCTTTGAACGCACATTGCGCCCGCCAGTATTCTGGCGGGCATGCCTGTCCGAGCGTCATTTCAACCCTCGAACCCCTCCGGGGGGTCGGCGTTGGGGATCGGCCACTCCCTCCTCTTTGGGGGCGGCCGGCCCCGAAATACAGTGGCGGTCTCGCCGCAGCCTCTCCTGCGCAGTAGTTTGCACACTCGCATCGGGAGCGCGGCGCGTCCAATGCCGTAAAACACCCAACTTTCTGAAATGTTGACCTCGGATCAGGTAGGAATACCCGCTGAACTTAAGC |

Accession number* - are sourced from the NCBI GenBank database.
