# Supplementary figures and images for: Bioprospecting and mechanistic insights of Trichoderma spp. for suppression of Ganoderma-induced basal stem rot in oil palm
Source: Front Nutr. 2025 Jul 10;12:1582047. doi: 10.3389/fnut.2025.1582047 (PMC12287078; doi:10.3389/fnut.2025.1582047)

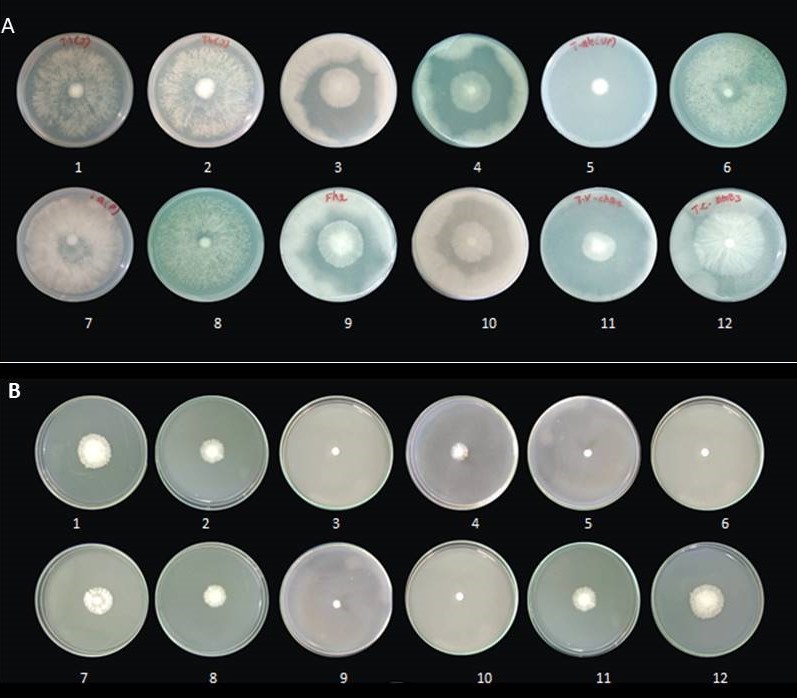

Supplement: Supplementary file 2 [file Image_1.jpeg]

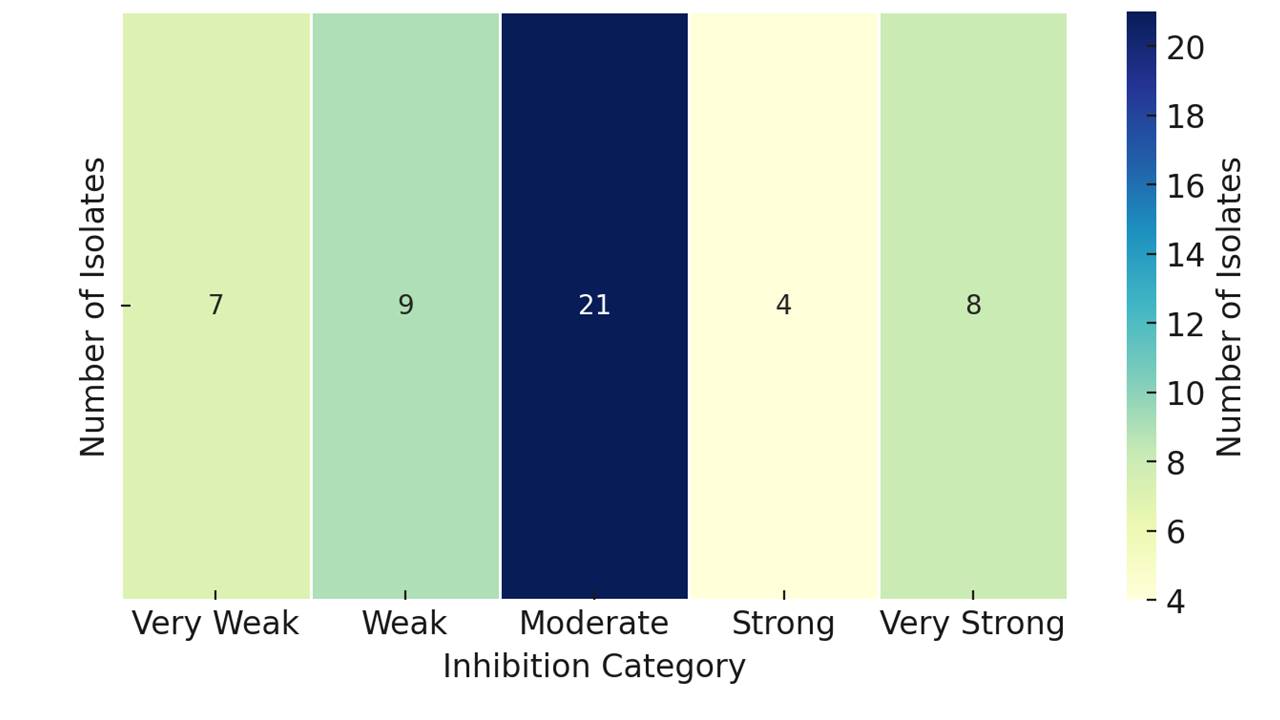

Supplement: Supplementary file 3 [file Image_2.jpeg]
